# Genome editing beyond transgenes: DNA-free technologies in crop improvement

**DOI:** 10.3389/fpls.2026.1942730

**Published:** 2026-10-05

**Authors:** Roshini Senthilkumar, Rakshana Palaniswamy, Sakthi Ambothi Rathnasamy, Veera Ranjani Rajagopalan, Kokiladevi Eswaran, Radhamani Sengodan, Ashok Kumar Govindasamy, Raveendran Muthurajan, Selvaraju Kanagarajan, Sudha Manickam

**Affiliations:** 1 Department of Plant Biotechnology, Centre for Plant Molecular Biology and Biotechnology, Tamil Nadu Agricultural University, Coimbatore, Tamil Nadu, India; 2 Department of Agronomy, Directorate of Crop Management, Tamil Nadu Agricultural University, Coimbatore, Tamil Nadu, India; 3 Department of Vegetable Science, Horticultural College and Research Institute, Tamil Nadu Agricultural University, Coimbatore, Tamil Nadu, India; 4 Department of Plant Breeding, Swedish University of Agricultural Sciences, Lomma, Sweden

**Keywords:** CRISPR/Cas, crop improvement, DNA-free genome editing, non-GMO, precision breeding, RNP delivery

## Abstract

Conventional and transgenic approaches to crop improvement are increasingly constrained by biosafety concerns, stringent regulatory frameworks, and limited public acceptance, necessitating new precision breeding methods. DNA-free genome editing using the CRISPR/Cas9 approach has emerged as a transformative approach that enables precise, targeted genetic modifications without the stable integration of a foreign gene (transgene). This strategy transiently introduces CRISPR/Cas9 ribonucleoprotein (RNP) complexes, or *in vitro* transcribed mRNA, into plant cells. This review focuses primarily on CRISPR/Cas-mediated DNA-free genome editing and its applications in crop improvement. After targeted genome cleavage, the editing elements are rapidly degraded, leaving only the desired genomic modification and resulting in plants indistinguishable from those developed through conventional breeding. To achieve targeted, DNA-free genome editing, researchers have developed numerous molecular platforms and delivery systems, such as protoplast transfection, biolistic bombardment, nanoparticle-based systems, and emerging pollen- and meristem-targeted systems, for major crop species. Researchers have successfully enhanced several notable traits, including disease and pest resistance, nutritional quality, abiotic stress tolerance, and agronomic performance, in cereals, vegetables, oilseeds, and perennial crops. Recent advancements, including base editing, prime editing, and multiplex gene editing, are further expanding the precision, efficiency, and scope of DNA-free genome editing. Overall, CRISPR/Cas-mediated DNA-free genome editing offers a route to crop improvement that avoids stable transgene integration, though its wider deployment depends on progress in regeneration, molecular validation, and regulatory convergence.

## 1 Introduction

Global food production is under growing stress due to rapid population growth, rising food demand, climate variability, land degradation, and losses from pests and diseases. In 2050, the world population will be around 9.7 billion, which means that food production must be increased by 50-70% to meet the demand for food security (Food and Agriculture Organization of the United Nations, 2017). At the same time, plant pests and diseases cost the world an estimated USD 220 billion annually in terms of economic losses (Food and Agriculture Organization of the United Nations, 2017) and also cause 20-40% of all crop losses worldwide annually. These challenges underscore the need for new breeding approaches that improve productivity, stress resistance, and sustainability while ensuring consumer acceptance. Crop improvement is now no longer limited to traditional selection and hybridization but has advanced to molecular breeding and genome editing technologies. Conventional breeding and induced mutagenesis have greatly contributed to yield improvement, enhanced resistance to biotic and abiotic stresses, and crop adaptation to a variety of agro-climatic zones. These methods, however, are time-consuming and rely on a narrow range of genetic variability, leading to linkage drag and low efficiency in improving complex polygenic traits (Acquaah, 2012; Tester and Langridge, 2010). Induced mutagenesis using physical and chemical mutagens has increased genetic variation, producing useful alleles for increased yield, stress tolerance, and disease resistance. However, when natural or induced variation is insufficient, breeders sometimes use transgenic technology to directly transfer selected genes into crop genomes (Raman, 2017). A significant step in agricultural biotechnology has been the introduction of genetically modified organisms (Qaim and Kouser, 2013). Golden Rice was developed to address vitamin A deficiency by expressing β-carotene in the endosperm (Ye et al., 2000), and herbicide-tolerant soybean (Guo et al., 2020). These examples illustrate the potential of transgenic solutions to agricultural problems where conventional breeding cannot find enough variation. However, they can lead to unintended genetic consequences and may cause problems with allergenicity, gene flow, pest resistance, weed development, and impacts on non-target organisms (Nap et al., 2003). As a result, strict biosafety measures, moral considerations, and social and economic concerns have restricted the use of transgenic crops. Site-directed nucleases (SDNs), such as zinc finger nucleases (ZFNs), transcription activator-like effector nucleases (TALENs), and CRISPR/Cas nucleases, enable precise, targeted genome modification. These technologies differ from traditional genetic engineering, which produces mutations that may be indistinguishable from natural genetic variation. The simplicity, efficiency, and programmability of CRISPR/Cas make it the most versatile and widely used platform (Jinek et al., 2012; Voytas and Gao, 2014). Examples of successful applications include powdery mildew-resistant wheat through MLO gene editing (Wang et al., 2014), and non-browning mushrooms through targeted editing of PPO genes (Waltz, 2016). Early applications of CRISPR/Cas-mediated genome editing primarily relied on introducing DNA constructs carrying the editing machinery into plant cells via *Agrobacterium*-mediated transformation or particle bombardment. These techniques were effective, but led to temporary or permanent integration of recombinant DNA, thus bringing edited crops under GMO regulation in many countries (Metje-Sprink et al., 2019). To overcome these limitations, DNA-free genome editing has emerged as a revolutionary technology in plant biotechnology. This approach uses genome-editing tools such as CRISPR/Cas ribonucleoprotein (RNP) complexes or short RNA molecules that do not integrate into the plant genome (Woo et al., 2015). These components are then edited, and the extra DNA is naturally degraded, leaving only the desired DNA modification. The short-term expression of the nuclease diminishes insertional mutagenesis and off-target effects compared to the long-term expression of the nuclease that would otherwise occur (Zhang et al., 2016). Furthermore, some regulatory bodies have suggested that plants edited without foreign genetic material may not be considered GMOs, depending on the editing method and the genetic changes introduced (Turnbull et al., 2021; Wolt et al., 2016). This review offers a comprehensive perspective on CRISPR/Cas based genome editing, covering diverse editing platforms, delivery technologies, molecular outcomes, crop-specific applications, and regulatory and commercialization opportunities. It also highlights recent advances in RNP-based editing and Cas12a-mediated editing, as well as emerging delivery strategies, including lipofection, pollen- and meristem-based delivery, and nanotechnology-based approaches. Importantly, the review provides a detailed discussion on the advances, challenges and limitations associated with each delivery and editing strategy. Overall, it provides an up-to-date perspective on the current state and prospects of DNA-free genome editing in crop improvement.

## 2 Advances in genome editing technologies

Genome editing technologies, especially the CRISPR/Cas system, have revolutionized plant biotechnology by enabling easy, efficient editing of specific genes with high precision and low cost (Chen et al., 2019; Jinek et al., 2012). These technologies rely on sequence-specific nucleases (SSNs) or site-directed nucleases (SDNs) that create double-strand breaks (DSBs) at specific genomic loci. Two primary pathways repair DSBs: Non-Homologous End Joining (NHEJ) and Homology-Directed Repair (HDR). NHEJ can fuse non-homologous DNA ends directly without a template and often generates small insertions or deletions (indels) that disrupt the gene. Conversely, HDR can introduce precise nucleotide substitution or targeted sequence insertions using an HDR repair template. These repair mechanisms can be exploited for targeted genome editing in plants. Depending on the kind of genetic modification added, genome editing strategies can be grouped into three categories: SDN-1, SDN-2 and SDN-3. SDN-1 employs NHEJ repair without a template and creates small indels which disrupt gene function. During HDR, SDN-2 employs a short repair template to introduce nucleotide substitutions or small sequence changes. Mutations generated by both SDN-1 and SDN-2 resemble natural variation and conventional mutagenesis. SDN-3 uses HDR to insert larger DNA fragments or transgenes. Researchers have created several programmable nucleases that cleave targeted DNA. ZFNs, one of the earliest genome editing tools, are made up of zinc-finger DNA-binding domains and the *Fok*I endonuclease that are linked together to recognize specific DNA sequences. ZFNs provided evidence for targeted mutagenesis without stable DNA integration, but their design and delivery complexity limited their use across a broader range of crops (Shukla et al., 2009). TALENs’ enhanced target specificity and design flexibility (TALE proteins from *Xanthomonas* bacteria fused to the *Fok*I nuclease) enabled efficient gene disruption in crops (Cermak et al., 2011). The first use of TALEN in rice achieved successful targeted mutagenesis, but DNA delivery remained the primary transfer method (Li et al., 2012). CRISPR/Cas is the most popular genome-editing technology because of its simplicity, efficiency, and programmability. It uses a guide RNA (gRNA) to direct the Cas nuclease to the target DNA region. Target recognition requires a short-conserved sequence next to the target site, known as a protospacer adjacent motif (PAM). The commonly used *Streptococcus pyogenes* Cas9 (SpCas9) recognizes the PAM sequence 5′-NGG-3′ (Hsu et al., 2014). CRISPR/Cas has been used to create powdery mildew-resistant wheat by editing *MLO* (Wang et al., 2014), non-browning mushrooms by removing polyphenol oxidase (Waltz, 2016), high-oleic soybean by editing *GmFAD2-1A* and *GmFAD2-1B* (Do et al., 2019) and aroma-enhanced rice by editing *BADH2* (Ashokkumar et al., 2020). Researchers have developed new nucleases and editing approaches, expanding the CRISPR toolbox. Cas12a (Cpf1) can target a variety of PAM sequences and creates staggered DNA breaks, expanding the range of DNA targets it can address. Base editing avoids the need to create DSBs, thus decreasing the risk of unintended mutations. To further improve precision, prime editing combines a Cas9 nickase with reverse transcriptase and a prime-editing guide RNA (pegRNA) to enable targeted insertions, deletions, or substitutions, eliminating the need for donor DNA templates (Anzalone et al., 2019). These innovations have significantly improved the precision, efficiency, and flexibility of genome-editing technologies for crop improvement (Lokya et al., 2025).

## 3 Transition towards transient and non-integrative genome editing strategies

In classical DNA-free genome-editing methods, primary transformants (T_0_ plants) are typically heterozygotes for the introduced transgene at the integration site. During subsequent generations, both the transgene and the edited genomic loci segregate independently according to Mendelian inheritance. Therefore, obtaining plants with the desired edit but without the transgene requires multiple generations of selfing, backcrossing, or segregation analysis, a laborious and time-consuming process. In addition, this process faces biological and technical challenges, including low *in vitro* transformation and regeneration efficiencies in many crop species, long generation times in woody and perennial plants, the genomic complexity of polyploid crops, and multiple-copy T-DNA integration, which further complicates the identification of transgene-free edited lines.

Increasing regulatory demands and public apprehensions about transgenic crops have spurred the adoption of genome-editing techniques that do not result in permanent DNA incorporation. This shift has enabled transient, non-integrative genome-editing methods. These techniques involve directly delivering genome-editing components such as CRISPR/Cas RNP complexes or messenger RNA into plant cells, enabling precise genetic alterations without introducing foreign DNA sequences. Transient genome editing ensures that the added editing machinery is active only briefly before endogenous cellular processes degrade it. This brief expression minimizes the possibility of insertional mutagenesis and reduces off-target consequences linked to prolonged nuclease activity.

## 4 Concept of genome editing without genetic footprints

Genome editing that leaves no genetic traces represents the pinnacle of development in crop improvement techniques. In this approach, researchers make specific genetic modifications while ensuring that no residual editing tools, selectable markers, or foreign nucleic acids remain in the plant’s final genome. These “footprint-free” or “clean” edits closely mimic spontaneous or naturally occurring mutations and align with precision breeding (Chen et al., 2024; Gao, 2021). Genome editing that leaves no genetic traces addresses major biosafety and regulatory concerns while also improving public acceptance of modern plant biotechnology. As crop enhancement increasingly prioritizes sustainability, climate adaptability, and rapid varietal development, footprint-free genome editing is becoming an essential technology for responsible and advanced plant breeding (Chen et al., 2024).

To rule out unwanted insertions or persistent editing components, verification usually includes PCR/qPCR and, when necessary, whole-genome sequencing (Gao, 2021). Compared with sustained expression from DNA vectors, the transient nature of the reagents also limits nuclease activity, reducing the risk of off-target breakage (Chen et al., 2024).

DNA-free editing has three interconnected effects on crop biotechnology. First, it improves biosafety by reducing off-target risk by limiting nuclease exposure and decreasing the likelihood of unwanted insertions (Gao, 2021; Zhang et al., 2018). Second, it can streamline regulatory pathways: several jurisdictions have indicated that edits produced without recombinant DNA may face less onerous regulation than transgenic events, depending on edit type and detection of foreign sequences. Third, breeders who want to respond quickly to biotic and abiotic stresses can benefit from its ability to deploy traits into elite cultivars without lengthy backcrossing to eliminate transgenes. Even with these benefits, challenges remain. Many species still struggle with efficiently delivering genome-editing components into plant cells capable of regeneration. Furthermore, standardized molecular assays are needed to conclusively show that foreign DNA is not present. Additionally, regulatory practices continue to differ among countries, and clear proof of the product’s advantages and safety will be necessary for public adoption (Metje-Sprink et al., 2019; Turnbull et al., 2021).

## 5 DNA-free genome editing platforms and molecular strategies

In the last 10 years, genome-editing technologies have revolutionized the study and enhancement of plant biology. The most commonly used platform for precise, efficient genome modification across a variety of plant species is the CRISPR/Cas9 system (Bortesi and Fischer, 2015; Zhang et al., 2018). Early CRISPR applications used plasmid DNA encoding the editing machinery, delivered via *Agrobacterium* or biolistic methods (Altpeter et al., 2016). While effective, these DNA-based methods also pose biosafety and regulatory challenges, including the potential for foreign DNA to integrate into plant genomes (Woo et al., 2015). An alternative approach uses DNA-free genome editing to overcome these limitations. These methods deliver genome-editing components in transient, non-integrating vectors such as synthetic oligonucleotides, *in vitro*-transcribed messenger RNA (mRNA), and preassembled CRISPR/Cas RNP complexes (Liang et al., 2015). The transient nature of these systems greatly reduces off-target mutagenesis and stable transgene integration, and makes it easier to distinguish gene-edited crops from transgenic plants for regulatory purposes (Zhang et al., 2016). Although it has great promise, DNA-free genome editing faces technical hurdles, including the plant cell wall, which prevents uptake of large biomolecules such as RNPs or mRNA (Laforest and Nadakuduti, 2022). Several methods have been developed to overcome this obstacle, such as protoplast transfection, particle bombardment, electroporation and nanomaterial-based systems. However, these methods vary in efficiency, species applicability, regeneration ability, and scalability. Thus, understanding their mechanisms is important to ensure effective, efficient editing and to extend their applications to other crop species. Alongside advances in genome-editing technologies, progress has been made toward DNA-free genome editing. Traditional CRISPR/Cas methods that use plasmid DNA or viral vectors may lead to continuous nuclease expression, increasing the risk of off-target changes and unwanted genomic modifications. DNA-free systems, by contrast, offer greater temporal control of nuclease activity, improving editing specificity and genomic stability. In plant biotechnology, where genetic integrity, environmental safety, and regulatory acceptance are crucial, these advantages matter most.

### 5.1 CRISPR/Cas RNP-mediated genome editing

The most well-studied platform for DNA-free genome editing in plants is CRISPR/Cas RNP complexes. These complexes are preassembled Cas9 protein with synthetic guide RNAs that are catalytically active and can cause targeted DNA DSBs upon delivery to cells. This approach was first shown to be feasible in rice, lettuce, Arabidopsis, and tobacco protoplasts, where efficient genome editing does not require introducing foreign DNA (Woo et al., 2015). CRISPR/Cas9 RNPs are composed of the Cas9 endonuclease and a guide RNA that is responsible for recognizing the target. The guide RNA can be a single guide RNA (sgRNA) that includes both parts of the guide RNA, or it may be two distinct RNA molecules: CRISPR RNA (crRNA) and trans-activating guide RNA (tracrRNA) (Jinek et al., 2012). The guide RNA (gRNA) uses Watson-Crick base pairing to sequence-specifically identify the target locus (protospacer), and Cas9 cleaves the DNA three base pairs upstream of the protospacer adjacent motif (PAM) (Anders et al., 2014). HDR or NHEJ repairs the resulting DNA breaks, which can create sequence modifications or targeted mutations. The transient nature of RNPs is a major advantage over DNA-based delivery systems. The time available for nuclease activity is typically only a few hours (24-48) after delivery, as the components tend to degrade rapidly, reducing off-target cleavage and increasing on-target editing efficiency (Liang et al., 2017). Because no DNA template is supplied, preassembled RNP delivery removes the principal route by which editing constructs integrate, and in several jurisdictions RNP-edited plants carrying only small indels are treated as non-GMO (Metje-Sprink et al., 2019; Woo et al., 2015). This applies to the delivered cargo itself; where plasmids, selectable markers, donor templates, or DNA-containing co-delivery reagents are used at any stage, the absence of introduced sequence should be demonstrated rather than assumed (Section 8.3). Optimization of guide RNAs and Cas9 proteins has facilitated further progress in RNP-mediated editing. Cas9 is usually expressed in bacteria and purified by affinity chromatography using an affinity tag (Liang et al., 2017). Chemical modifications of guide RNAs, such as 2′-O-methyl phosphorothioate substitutions, increase nuclease resistance and editing efficiency, and stabilizing agents like trehalose and glycerol improve protein stability during storage (Hendel et al., 2015; Liang et al., 2017). Moreover, high-fidelity Cas9 variants tailored via structure-guided mutagenesis have been generated that minimize off-target editing while maintaining editing efficiencies (Kleinstiver et al., 2016). Alternative CRISPR nucleases have added to the versatility of RNP-based genome editing. Cas12a (Cpf1) targets A/T-rich PAMs and introduces a staggered DNA break, expanding potential target sites and enabling unique repair outcomes. It also has intrinsic RNase activity that enables multiplex genome editing via CRISPR-array processing (Kim et al., 2017; Zetsche et al., 2015). RNP-based genome editing has proven effective in rice and maize. Zhang and co-workers introduced LbCas12a, LbCas12a-E795L, AsCas12a and AsCas12a Ultra as RNP complexes into rice protoplasts, achieving nearly 100% editing efficiency at three of the four target sites. The RNP-based approach also outperformed plasmid-based delivery at both 32 °C and 25 °C (Zhang et al., 2022). Similarly, Cas12a RNP-mediated editing allowed transgene-free multiplex genome editing in citrus, including the generation of long nucleotide deletions and inversions. This method successfully generated 10 edited lines without any off-target mutations and resulted in enhanced resistance to canker (Su et al., 2024). In maize, Dong and co-workers used particle bombardment to deliver Cas12a RNPs and regenerated edited plants with an editing efficiency of approximately 7% using mannose selection (Dong et al., 2021).

### 5.2 RNA-based transient genome editing approaches

Researchers developed transient expression methods to reduce the risk of stable integration of foreign DNA. Transient Expression of CRISPR/Cas9 DNA (TECCDNA) is one of them, in which plasmid DNA vectors encoding the Cas9 nuclease and single-guide RNA (sgRNA) are introduced using techniques such as protoplast transfection or particle bombardment. The plasmid DNA containing the Cas9 protein and sgRNA is transiently expressed to produce Cas9 protein and sgRNA, which assemble to form the CRISPR/Cas9 RNP complex that can induce targeted genome double-strand breaks. Cells then repair these breaks via pathways such as NHEJ, which can lead to specific mutations. However, using plasmid DNA also raises the risk of unwanted integration of foreign DNA into the plant genome, limiting the usefulness of this approach for producing entirely DNA-free edited plants. To address these shortcomings, researchers developed a better design: transient expression of CRISPR/Cas9 RNA (TECCRNA). In durum wheat, Zhang and his associates showed that transient expression of CRISPR/Cas9 RNA could generate homozygous mutants without any transgene insertions in the T_0_ generation (Zhang et al., 2016). In addition, Liang and his co-workers developed an *in vitro*-transcribed (IVT) RNA delivery system using particle bombardment culture that reduces the risk of random integration of CRISPR/Cas9 sequences and allows generation of foreign-DNA-free plants within 9–11 weeks (Liang et al., 2018). Recently, optimization of the untranslated region (UTR), poly(A) tail, and protamine coating of IVT mRNA enhanced the efficiency of knockout, A-to-G editing, and C-to-T editing in rice and wheat, compared with the delivery of plasmid (Qiu et al., 2025). CRISPR/Cas9 base editing using *in vitro*-transcribed nCas9-PBE mRNA and TaALS-P174 sgRNA, delivered by particle bombardment into immature wheat embryos, enabled the development of transgene-free, herbicide-tolerant wheat plants (Zhang et al., 2019).

## 6 Delivery methods for DNA-free genome editing

Effective delivery of genome-editing components into plant cells is crucial for successful DNA-free genome editing. Researchers have developed and refined various delivery techniques for a range of plant species and tissue types, each with specific benefits and drawbacks. The delivery routes discussed in this section trade cargo capacity against tissue integrity (**Figure 1**).

**Figure 1:**
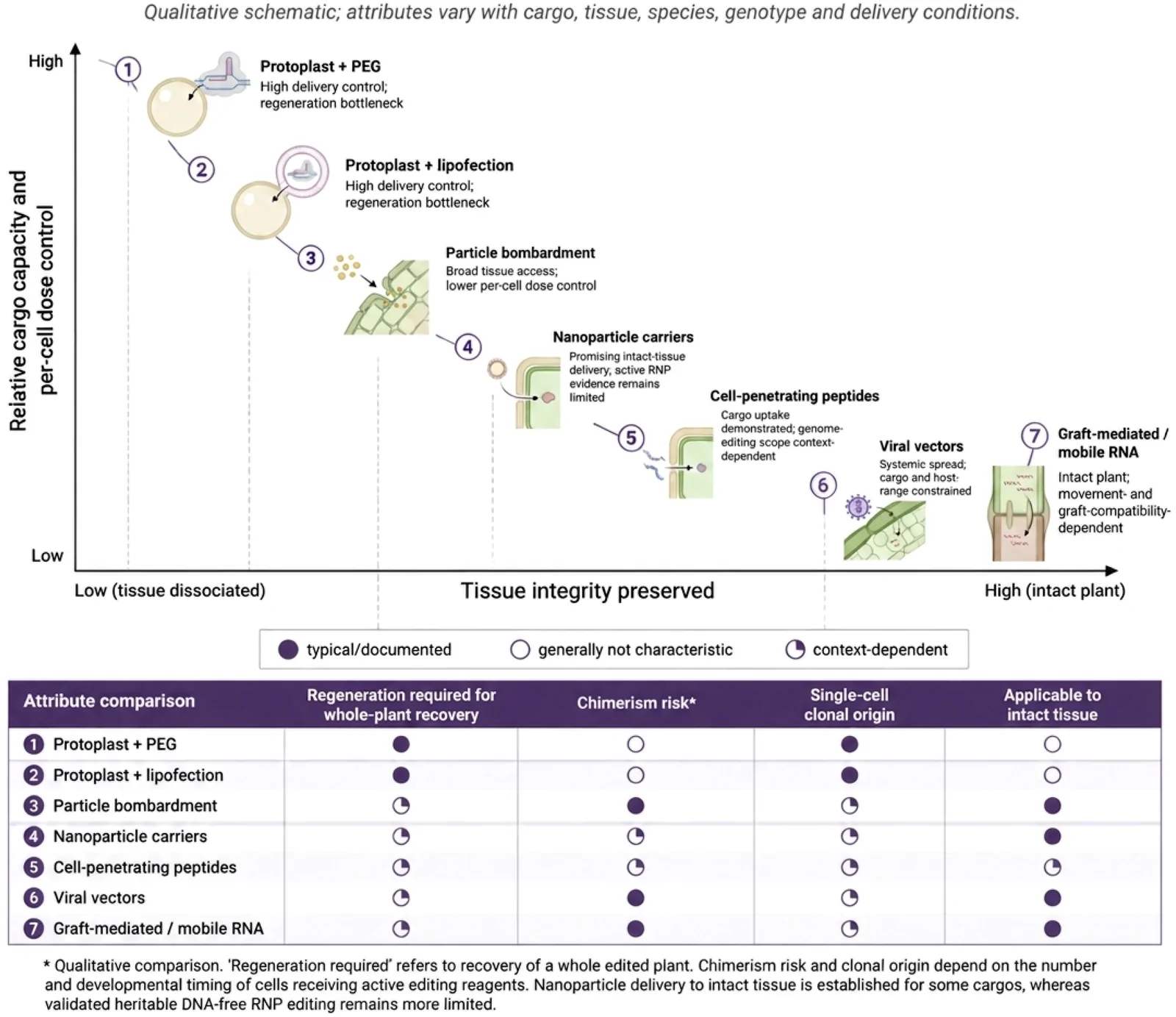
Delivery routes for DNA-free genome editing in plants: trade-offs between cargo delivery control and tissue integrity. Seven delivery routes are positioned qualitatively according to the degree of control over cargo composition and per-cell dose (vertical axis) and the extent to which tissue architecture is preserved (horizontal axis). Routes that dissociate tissue into single cells, namely polyethylene glycol (PEG)-mediated and lipofection-mediated delivery to protoplasts (1, 2), permit saturating and precisely metered delivery of preassembled ribonucleoprotein (RNP) complexes to individual cells, but require regeneration of whole plants from single protoplasts. Particle bombardment (3) reaches intact explants but delivers unevenly between cells. Nanoparticle carriers (4) and cell-penetrating peptides (5) are applicable to intact tissue, although cargo capacity is constrained by cell-wall penetration and intracellular release. Viral vectors (6) and graft-mediated transfer of mobile RNA (7) preserve the intact plant but are limited by cargo size, host range, and dependence on movement or graft compatibility. The accompanying matrix summarizes four attributes for each route: filled circles denote attributes that are typical or documented, open circles those that are generally not characteristic, and half-filled circles those that depend on implementation. The inverse relationship between the two axes reflects a general constraint of current delivery technology rather than a property of any individual method, and no route presently combines single-cell dose control with intact-tissue applicability. Created in BioRender. Kanagarajan, S. (2026) https://BioRender.com/z5h7gbj.

### 6.1 Protoplast-mediated genome editing

Protoplast transformation is the most common method for delivering DNA-free editing components into normal plant cells. Enzymatic digestion of cell walls with cellulase and pectinases produces protoplasts that expose the plasma membrane, allowing efficient uptake of macromolecules (Yoo et al., 2007). Eliminating the cell wall barrier significantly improves delivery of RNP complexes and mRNA, making protoplasts an appropriate system for genome editing (Woo et al., 2015). The isolation procedure must be carefully adapted to each plant species and tissue type (**Figure 2**). Leaf mesophyll cells are particularly convenient because they are plentiful and easy to isolate. Digestion is usually carried out in osmotically balanced solutions with mannitol or sorbitol to prevent protoplast lysis for 2 to 16 hours, depending on tissue and species (Woo et al., 2015). The protoplasts are purified by density gradient centrifugation or filtration to remove debris and ungerminated material. Transient permeabilization of the membrane is achieved by PEG-mediated uptake or electroporation for RNP transfection. Protoplasts are incubated with the editing components, high-molecular-weight PEG (4000–6000 Da), and calcium ions to promote membrane fusion (Yoo et al., 2007). In general, PEG concentrations range from 20–40% and incubation periods from 10 to 30 minutes, but efficiency depends on PEG concentration, incubation time, and temperature (Ferrero et al., 2026). This milder method results in high viability but may be less efficient in some species than electroporation. PEG has been used in a range of model organisms and common crops, but it can be cytotoxic and contaminate downstream analysis (Subburaj et al., 2022). To address these drawbacks, recent research has focused on refining PEG dosage and alternative delivery methods such as lipofection and electroporation (Ferrero et al., 2026; Subburaj and Agapito-Tenfen, 2023). Edited protoplasts can be regenerated into whole plants under controlled tissue culture conditions, although regeneration efficiency depends on species and genotype (Reed and Bargmann, 2021). Several crops have been successfully edited by protoplast-based RNP delivery and regeneration. Rice protoplasts are particularly tractable, resulting in efficient editing and regeneration of fertile plants (Kim et al., 2017). The study developed an early-stage protoplast culture system for *Cannabis sativa*, addressing the major bottleneck of the lack of a reliable protocol for regenerating isolated protoplasts into complete plants (Král et al., 2025). The authors tested different donor tissues and culture conditions and identified 1-2-week-old leaves from *in vitro*-grown seedlings as the most suitable source for protoplast isolation. However, the study achieved only partial regeneration rather than complete plant regeneration. Therefore, it provides a foundation for future C*annabis* protoplast-to-plant protocols and potential applications in genome editing, transformation, and biotechnology, but further optimization is still required. Recent advances in protoplast-mediated CRISPR/Cas9 RNP delivery have enabled DNA-free genome editing across diverse crops, although editing efficiencies vary considerably depending on the species, target gene, and delivery conditions. In carnation, direct delivery of CRISPR/Cas RNP complex into protoplasts resulted in mutation frequencies of 8.8–10.8% for *ACO1* and 0.2–58.5% for *ACS1* (Adedeji et al., 2024). In cabbage, protoplast RNP delivery achieved 25% indel frequency using a 1:10 Cas9-to-sgRNA molar ratio combined with a short incubation period (Lee et al., 2024).

**Figure 2:**
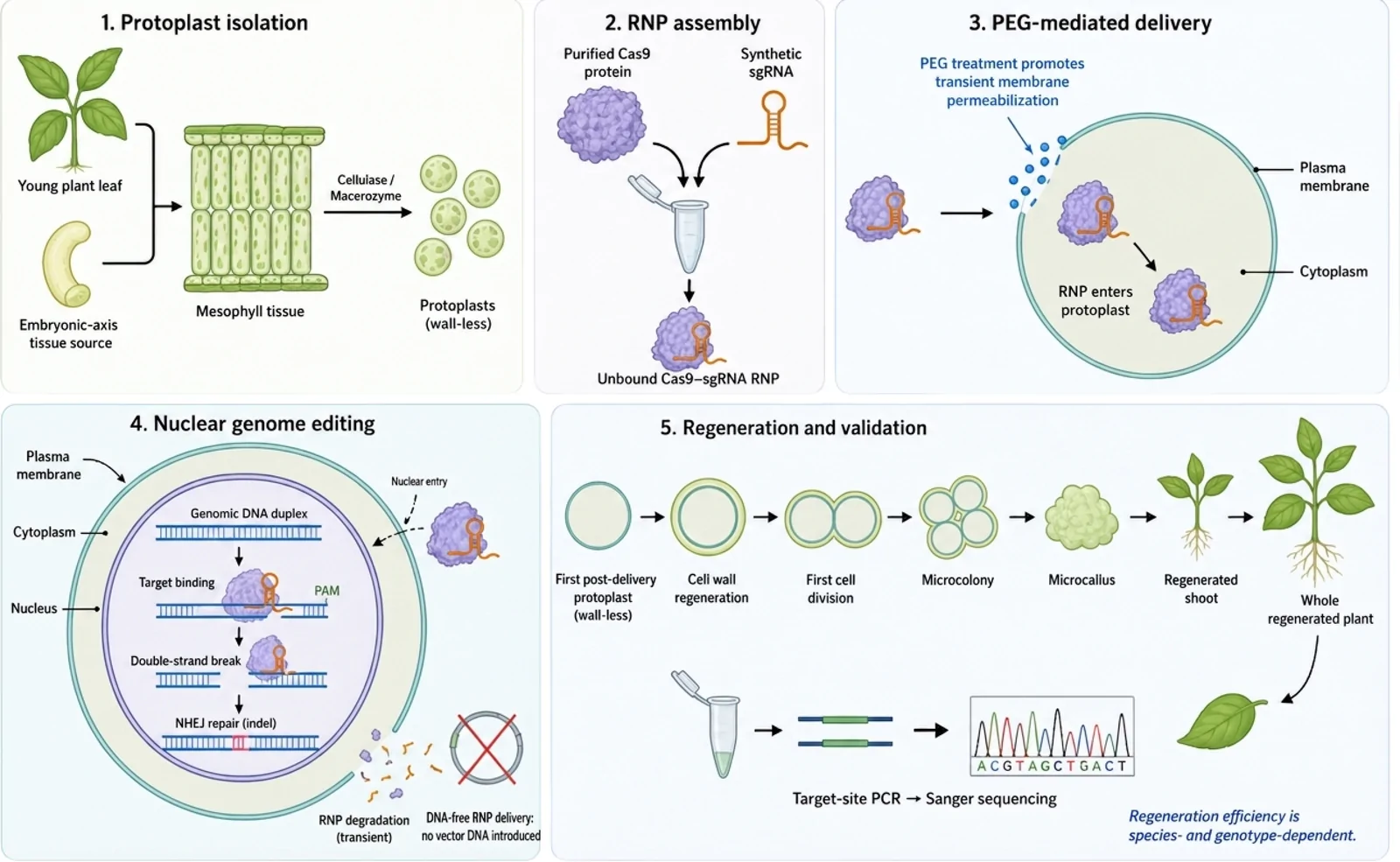
DNA-free CRISPR/Cas9 ribonucleoprotein delivery, genome editing, and plant regeneration from protoplasts. Plant protoplasts are isolated from leaf mesophyll tissue or embryonic-axis-derived tissue by enzymatically degrading the cell wall with cellulase and macerozyme, yielding spherical, wall-less cells (Stage 1). Purified Cas9 protein is assembled in vitro with a synthetic single-guide RNA (sgRNA) to generate an unbound Cas9–sgRNA ribonucleoprotein (RNP) complex, without use of plasmid DNA, donor DNA, or other vector DNA (Stage 2). Polyethylene glycol (PEG) treatment transiently promotes plasma-membrane permeabilization and enables uptake of the preassembled RNP into the protoplast cytoplasm (Stage 3). Following nuclear entry, the RNP recognizes a genomic target adjacent to a protospacer-adjacent motif (PAM), induces a double-strand break, and repair through non-homologous end joining (NHEJ) produces small insertions and/or deletions (indels) at the target site (Stage 4). The RNP is transient and is degraded after editing; because the cargo is delivered as a protein–RNA complex, no vector DNA is introduced. Edited protoplasts subsequently regenerate a cell wall, divide, form microcolonies and microcalli, and can regenerate shoots and whole plants. Target-site edits are confirmed by PCR amplification followed by Sanger sequencing (Stage 5). Regeneration competence, editing efficiency, and recovery of edited plants depend on species, genotype, tissue source, and culture conditions. Created in BioRender. Kanagarajan, S. (2026) https://BioRender.com/xv98i5t.

The preference for protoplasts over other cell systems in DNA-free editing rests on three properties. First, removing the wall permits delivery of a saturating dose of pre-assembled nuclease to a single cell, rather than the compromises on cargo size, dose, or tissue integrity that every intact-tissue route must accept (Section 7.1). Second, because each regenerant derives from a single edited cell, protoplast-derived plants are non-chimeric, and their edited alleles segregate in a Mendelian manner, in contrast to explant- and meristem-based systems in which only a fraction of cells receive the cargo and mosaicism is an intrinsic outcome (Lin et al., 2018); this point is developed further in Section 8. Third, the same protoplast population used for regeneration can first-rank guide RNA designs *in vivo* in the target genotype, so only validated guides enter the regeneration pipeline (Sahab et al., 2024). The delivery advantage is evident in the comparison reported by Zhang and his co-workers, in which Cas12a RNP delivery to protoplasts approached complete editing at three of four target sites, while plasmid delivery of the same reagents was substantially less effective (Zhang et al., 2022). These advantages are purchased at the cost of the regeneration bottleneck discussed in Section 7.2, which remains the principal constraint on the approach.

### 6.2 Particle bombardment-based delivery

In biolistic delivery, the plant cell wall is physically perforated with high-velocity gold or tungsten microprojectiles coated with RNP complexes, and direct editing of intact tissues, such as immature embryos and meristems, is not possible by isolating protoplasts (Laforest and Nadakuduti, 2022). This method is especially useful for species that are difficult to infect with *Agrobacterium* or ones where protoplasts cannot be cultured (Laforest and Nadakuduti, 2022; Poddar et al., 2023). The optimization of physical parameters like helium pressure, target distance, and gold particle size plays a crucial role in maximizing the delivery efficiency and minimizing tissue damage, which is essential for the success of the treatment (Ma et al., 2024; Tanaka et al., 2022). Stable transformation frequencies were significantly higher in wheat when ruptured disks were used with 0.6 μm gold particles and the pressure of disks optimized (Tanaka et al., 2022). Although widely used, biolistic delivery without selection markers often yields editing rates under 10%, and the physical effects of bombardment can damage the genome and create chimerism (Ramakrishnan et al., 2026; Rocha et al., 2025).7

### 6.3 Electroporation approaches

Using short electric pulses, electroporation creates temporary holes in cellular membranes that allow macromolecules such as proteins and nucleic acids to enter. In addition to being commonly used for protoplast transformation, the physical delivery technique has demonstrated potential for delivering editing components to specific intact plant tissues (Sheen, 2001). By applying an external field, the mechanism depolarizes and reorganizes lipid bilayers, resulting in aqueous pores that allow charged molecules to pass through (Neumann et al., 1982). To balance cell viability and delivery efficiency, electroporation parameters must be carefully optimized for plant protoplasts. Key parameters include electric field strength, pulse duration, pulse waveform, and the number of pulses. The composition and ionic strength of the electroporation buffer can also influence delivery efficiency and cell viability. Electroporation has been used to deliver CRISPR/Cas9 RNP complexes into plant protoplasts, providing a DNA-free approach for genome editing. In cabbage (*Brassica oleracea*) protoplasts, electro-transfection with CRISPR/Cas9 RNPs enabled targeted mutagenesis of the *PDS1* gene and resulted in higher mutagenesis and colony-formation efficiencies than PEG-mediated transfection (Lee et al., 2020). Neon electro-transfection of Cas9 RNPs into soybean protoplasts was found to be effective; the internalization of GFP-tagged Cas9 was observed under the microscope (Subburaj and Agapito-Tenfen, 2023). In the same study, a 10 ms pulse duration was optimal; however, cell viability and initial cell division decreased as voltage increased. The proportion of indels increased with voltage, and at 1,300 V achieved 3.8-8.1% indels in CPR5 target sites, demonstrating electroporation as an alternative to PEG to introduce indels without DNA (Subburaj and Agapito-Tenfen, 2023). Simple 24–28 V batteries deliver CRISPR RNP complexes and plasmid vectors into intact faba bean embryos, enabling genome editing without tissue culture (Augustine et al., 2025). Two needles attached to the battery deliver current to permeabilize cell walls and membranes, allowing macromolecule entry, while a droplet of RNP or plasmid is applied to the embryo (Augustine et al., 2025). This was validated by stable GFP expression, integration of the *hpt* marker, and detection of the DsRed marker (Augustine et al., 2025). This method achieved 50% mutation efficiency at the PDS locus in faba bean, which enables the production of chimeric albino mutants in 7–8 months, and it is applicable to both leaf and embryo tissue, with little training required and without the need for specialized equipment (Augustine et al., 2025). In contrast to traditional plant electroporation, cabbage protoplasts only required 1000 V for 3.4% editing efficiency, while soybean protoplasts required 500–1300 V pulses (Ramakrishnan et al., 2026). This is a single-locus, single-species proof of concept and more widespread validation is required (Augustine et al., 2025). It also reflects a broader trend toward low-cost, portable electroporation; the OpenPore system achieves commercial transfection efficiency with 9 V/1.5 V batteries in mammalian cells (Nesmith et al., 2026) and uses nanoneedle arrays for direct intranuclear RNP delivery in dendritic cells.

### 6.4 Emerging delivery strategies

#### 6.4.1 Nanoparticle-based delivery

Nanomaterial-mediated delivery is a developing approach for plant genome editing without DNA. Researchers have tested several nanomaterials to deliver proteins or nucleic acids to plant cells, including mesoporous silica nanoparticles, carbon-based nanomaterials, gold nanoparticles, lipid-based carriers, and cell-penetrating peptides, but evidence of their efficacy varies widely by cargo, nanomaterial, and plant tissue type. They may offer benefits such as cargo protection, improved cellular uptake, and less reliance on the traditional transformation and tissue culture methods. Successful delivery of a reporter or non-CRISPR cargo, however, does not guarantee efficient delivery of an active Cas9-sgRNA RNP complex or heritable genome editing (Demirer et al., 2021). An example of this distinction is provided by cell-penetrating peptides. An R9-cys-mCherry construct was reported to enter *Arabidopsis* root tips, leaves, and microspore material from *Brassica*; 100% of the root tips were transfected with reporter protein, but not with CRISPR-mediated genome editing. Therefore, these results indicate the ability of cell-penetrating peptides to deliver proteins into cells (Wu et al., 2021). Other nanocarriers, such as mesoporous silica, continue to offer great potential because their surface properties and interactions can be tailored for controlled delivery. However, penetration of the plant cell wall, tissue specificity, intracellular trafficking, cargo release, potential toxicity, and regeneration remain important engineering and biological limitations. The term “transgene-free” should be limited to studies which directly demonstrate delivery of DNA-free components of the CRISPR system, editing targeted genes, and the absence of vector-derived sequences, and, if applicable, the transfer of the edit to offspring (Demirer et al., 2021). Reviews have noted the potential of intact RNP delivery and transgene-free editing, but quantitative experimental data on editing efficiencies in plant species and independent validation remain limited (Vats et al., 2022). In woody species, nanoparticle-based RNP delivery remains largely untested, while PEG-mediated protoplast transfection and particle bombardment are the most reported methods (Ramakrishnan et al., 2026). Similarly, nanocarrier-based delivery is largely limited to controlled experimental systems, toxicity, scalability, and field translation hinder the routine use of these carriers in breeding (Singh et al., 2026). In summary, nanoparticles offer promising possibilities for RNP delivery; however, sufficient experimental evidence does not yet support this potential (Demirer et al., 2019).

#### 6.4.2 Pollen and meristem targeted delivery

Haploid-inducer-mediated genome editing (HI-Edit, also termed IMGE) uses a haploid inducer line carrying the CRISPR/Cas9 construct as a pollen donor, so that editing components act transiently on the maternal genome before the paternal chromosomes are eliminated, yielding edited haploid progeny free of the transgene. Doubling gives homozygous lines without tissue culture or transgene segregation (Kelliher et al., 2019). By targeting meristematic cells and reproductive tissues, researchers can achieve heritable genome edits without traditional transformation and regeneration. This strategy is justified by the idea that editing events that take place in meristem stem cells or germline cells will either contribute to the production of chimera plants or be passed on to offspring (Zhao et al., 2016). Researchers have investigated delivery of editing components to these specialized tissues using a variety of techniques. Pollen magnetofection uses magnetic nanoparticles and an external magnetic field to drive cargo into pollen grains. Researchers demonstrated the method by delivering plasmid DNA into cotton pollen, which, upon pollination, produced transgenic offspring (Zhao et al., 2017); adapting it to RNP cargo, and thus to DNA-free editing, remains prospective. This method may allow quick production of modified plants without tissue culture, though it is technically difficult because of the protective pollen wall (Zhao et al., 2016). Another method for accomplishing germline editing is the direct injection of editing components into developing ovules or floral meristems. Rocha and coworkers expanded this strategy to woody perennials by biolistically delivering CRISPR/Cas9 RNPs into the shoot apical meristems of citrus and the axillary meristems of poplar, resulting in targeted edits in tissues where plasmid delivery was unsuccessful, although chimeric events were still observed (Rocha et al., 2026).

#### 6.4.3 Lipofection-mediated transfection

Lipofection is a lipid-based delivery method used to introduce nucleic acids or RNP complexes into cells via lipid vesicles (liposomes). For DNA-free genome editing, the preassembled CRISPR/Cas9 RNP is delivered directly into plant protoplasts without plasmid gene intervention (lipofection). The positively charged lipids combine with the negatively charged nucleic acids or RNPs; this fusion with the cell membrane allows intracellular release of the editing components.

Once inside the cell, the Cas9-sgRNA complex induces targeted double-strand breaks, which endogenous DNA repair pathways, including NHEJ, repair, leading to genome editing. Because the delivered molecules (protein and RNA) are transitory (due to rapid degradation), the transgene of interest can be short-lived and eliminated. Lipofection has already succeeded in protoplast systems and is a viable approach for producing transgene-free, genome-edited plants, especially in species that support protoplast regeneration (Liang et al., 2017; Woo et al., 2015). Cationic lipids also prepare RNP complexes with CRISPR components that are delivered into plants via protoplasts, where they mediate stable, integration-free DNA editing without transient electroporation or electrofusion. Lipofection is gaining traction as a suitable platform for RNP delivery into plant protoplasts. In tobacco BY-2 protoplasts, Lipofectamine 3000 achieved a Cas9-RNP delivery efficiency of up to 66%, while RNAiMAX delivered up to 48% (Ramakrishnan et al., 2026). In the recalcitrant grapevine cultivar Nebbiolo, delivery of RNP using the lipofectamine method resulted in higher editing efficiency (11.3%) and no detectable foreign DNA compared to the PEG method, which resulted in a low editing rate and high foreign DNA contamination (Gambino et al., 2024). Although lipofection is a promising but less established method for plant editing than PEG or biolistic delivery in many woody species, in citrus, RNP transfection with lipofectamine resulted in plants edited without adding transgenes (Ramakrishnan et al., 2026).

#### 6.4.4 Viral vector-mediated DNA-free genome editing

To overcome these limitations, engineered RNA viruses are an emerging platform for DNA-free genome editing, as they can deliver CRISPR/Cas or gRNA components systemically without integrating foreign DNA into the plant genome. They can be efficiently replicated and spread systemically throughout plants, enabling high-frequency editing throughout the plant (Uranga et al., 2021; Uranga and Daròs, 2023). Likewise, engineered tomato spotted wilt virus (TSWV) vectors were also used to deliver Cas9, Cas12a, and base editors to various plant species, and base editing was achieved in up to 77.9% of regenerated pepper plants using the biocontained TSWV (Liu et al., 2023). Positive-strand RNA viruses provide different platforms for alternative cargoes (Uranga et al., 2021). A two-virus system delivered TEV-compatible Cas12a and PVX-compatible guide RNAs, enabling DNA-free genome editing without pre-existing Cas9 transgenic plants (Uranga et al., 2021). Compact RNA-guided nucleases can overcome viral cargo limitations. The compact ISYmu1 nuclease was delivered by TRV and enabled transgene-free germline editing in Arabidopsis, and an engineered eTnpBc variant achieved up to 90% somatic editing and complete editing in mutant progeny (Nagalakshmi et al., 2026). More recently, TRV-ISYmu1 was used with in planta shoot regeneration to obtain virus-free homozygous tomato mutants in one generation without tissue culture (Liu et al., 2026). A major drawback of viral editing is limited access to shoot apical meristems, which may limit the extent of germline transmission. Fusing mobile RNA elements, such as FT- or tRNA-derived sequences, to guide RNAs can help move editing signals into meristematic tissues (Uranga and Daròs, 2023). To simultaneously target three homoeoalleles and produce virus-free progeny, BYSMV-mediated delivery of Cas9 mRNA and sgRNAs to axillary meristems was employed in wheat (Qiao et al., 2025).

#### 6.4.5 Graft-mediated DNA-free genome editing

Recently, grafting has emerged as a new method to deliver genome-editing reagents, bypassing the limitations of tissue culture, transgene segregation, and viral vectors. The latter is an approach that involves incorporating tRNA-like sequences (TLS) in a guide RNA transcript and Cas9, with the aim of allowing long-distance delivery of the guide RNA/Cas9 editing machinery to a genetically different non-transgenic scion through the vasculature from a genetically transgenic donor rootstock. Yang and his co-workers demonstrated this graft-mobile CRISPR/Cas9 system enabled heritable, transgene-free edited progeny in *Arabidopsis thaliana* and *Brassica rapa*, without resorting to transgene segregation, tissue culture recovery, selection, or viral-vector delivery (Yang et al. 2023). Awan and his associates independently confirmed the key result of tRNA-tagged variants of CRISPR reagents moving from rootstock to scion and creating targeted mutagenesis at the scion-distal site (Awan et al. 2023), and Zaman and his co-workers similarly report that mobile CRISPR RNAs are a common feature of graft-transmissible editing (Zaman et al. 2023). This “graft-mobile” approach is especially relevant for species that are difficult to regenerate *in vitro* and vegetatively propagated, or for highly heterozygous crops, in which conventional transgene segregation by selfing is impractical or would result in undesirable genotypes (Simoni et al., 2025). The approach is nonetheless promising but still in its infancy stage; however, as the only experimental demonstration to date reports low and variable editing efficiency, as well as a lack of scalability beyond model systems, graft incompatibility between donor and recipient genotype is a significant hurdle for wider application of the technique. To improve the mobility and translational efficiency of CRISPR components in distal scion tissues, smaller Cas effectors, such as miniature Cas12, TnpB, or Fanzor, have been proposed. Furthermore, combining mobile CRISPR systems with inducible promoters, including heat-inducible promoters, could provide temporal control of editing activity in recipient tissues (Simoni et al., 2025).

### 6.5 Comparative evaluation of methods of DNA-free delivery

Although various delivery systems facilitate DNA-free genome editing, no single technique is universally suitable for every crop species and tissue type. Protoplast-based methods utilizing PEG or electroporation can efficiently introduce RNPs and mRNA; however, their success is heavily reliant on the viability of protoplasts and their ability to regenerate. Particle bombardment allows introduction of editing materials into whole tissues and challenging crops, but editing efficiency and material distribution can vary, and it may damage tissue. Nanoparticle- and cell-penetrating peptide-based systems offer promising alternatives to address cell-wall challenges and reduce reliance on tissue culture, but their effectiveness, consistency, scalability, and long-term biological impacts still need thorough assessment. Approaches targeting pollen, meristem, and other tissues may enable heritable editing without traditional regeneration, although challenges such as mosaicism and delivery to germline cells persist. Therefore, when assessing the appropriateness of a DNA-free delivery method, consider editing efficiency, cell or tissue viability, regeneration capacity, heritability, genotype specificity, scalability, reproducibility, and the ability to produce stable edited plants. **Table 1** provides a comprehensive overview of the delivery methods discussed in this section.

**Table 1:** A comprehensive overview of the various delivery methods employed for DNA-free genome editing in plants.

Method	Description	Editing efficiency	Significance	Limitations	References
Protoplast-Mediated Delivery	Introduces CRISPR cargo usually as RNPs into cell-wall free plant cells.	~100% at some rice/citrus Cas12a targets	Faster editor testing and direct delivery of CRISPR components	Protoplast regeneration remains a major bottleneck	(Ramakrishnan et al., 2026; Zhang et al., 2022)
PEG-Mediated Delivery	Chemical transfection of protoplasts using polyethylene glycol (PEG)	22.9-24.2% in sweetpotato; ~60% in eggplant	Simple, inexpensive and broadly accepted	Cytotoxicity and strong genotype dependence	(Brown et al., 2026; Ferrero et al., 2026)
Electroporation	Electric-pulse induced membrane permeabilization	3.8-8.1% in soybean	PEG free alternative with controllable delivery conditions	Protoplast viability drops during high voltage treatment	(Subburaj and Agapito-Tenfen, 2023)
Pollen/Meristem-Mediated Delivery	Direct targeting of reproductive tissues or shoot apical meristems	Pollen magnetofection achieves 2-12% transformation efficiency.	Potential for tissue culture free genome editing	Low viability, chimerism, limited validation	(Alam, 2026)

## 7 Challenges in delivery to intact plant tissues

### 7.1 The cell wall as a physical barrier

The plant cell wall is the most significant barrier differentiating plant delivery from mammalian delivery. Primarily made of cellulose, hemicellulose, and pectin, it acts as a size-exclusion barrier that limits the passive passage of large macromolecular structures such as Cas9–sgRNA RNP complexes (approximately 160 kDa). The cell wall is largely impermeable to pre-assembled RNPs and the nucleic acids that encode them (Ramakrishnan et al., 2026). Although plasmodesmata channels connect adjacent cells and there are open pores in younger tissues, these characteristics cannot be consistently utilized for uniform, targeted delivery throughout various types of heterogeneous tissues. Woody plants pose an additional challenge because their high levels of structural polymers like lignin and cellulose hinder both cargo entry and the enzymatic breakdown needed for protoplast extraction (Scintilla et al., 2021). As a result, any delivery method must either physically circumvent the cell wall or adapt the cargo to cross it on its own.

### 7.2 Constraints prevailing in protoplast mediated delivery

Protoplast transfection, which involves enzymatic removal of the cell wall followed by introduction of editing reagents via PEG or electroporation, is the most widely used method for DNA-free genome editing because PEG-mediated fusion enables efficient targeting. This technique has been applied effectively to various species, including potato, tomato, grapevine, and rapeseed. Nonetheless, substantial limitations remain when the objective is editing in whole tissues. One primary challenge in species like cultivated tomato is the difficulty of shoot regeneration from transfected protoplasts (Liu et al., 2022). Regenerating entire plants from protoplasts is technically difficult and varies significantly between species, so its use is currently limited to species with effective protoplast regeneration systems. Additionally, the enzymatic digestion needed to generate protoplasts may trigger stress responses and modify gene expression patterns, which could jeopardize the accuracy of subsequent editing and the resulting phenotypic characteristics (Reed and Bargmann, 2021).

A further constraint, less often stated in the DNA-free literature, is that the regeneration step itself is mutagenic. Whole-genome analysis of potato regenerants found that all fifteen plants recovered from protoplasts carried aneuploidy or structural chromosomal change, compared with 18% of regenerants from stem explants used for *Agrobacterium*-mediated transformation and none among cutting-propagated controls; resampling different leaves of the same plant gave different copy-number profiles, indicating that instability persists through development rather than resolving at regeneration (Fossi et al., 2019). At the nucleotide level, whole-genome sequencing of regenerated rice revealed a base-substitution rate of 1.74 × 10^−6^ per site per regeneration, approximately 248-fold higher than the spontaneous rate reported for Arabidopsis, alongside numerous indels and retrotransposon activation (Miyao et al., 2012). Culture-induced change is also epigenetic, with DNA methylation patterns markedly less stable in regenerants than in seed-grown plants (Kaeppler et al., 2000). The agronomic consequences are measurable: all 54 chrysanthemum protoplast regenerants assessed under commercial production differed significantly in at least one of flower number, flower size, or plant size (Eeckhaut et al., 2020), and in strawberry, fruit yield was significantly reduced in 15 of 21 protoclones evaluated in the field (Barceló et al., 2019). Researchers recognized this phenomenon early in oilseed rape protoplast regenerants and gave it the designation “proto-variation” (Jain et al., 1988).

Ploidy status offers a practical early filter. Across five pea genotypes, protoplast-derived calluses differed markedly in proliferation and regeneration competence both between and within genotypes, and flow cytometry showed these differences to be correlated with endoreduplication; fertile plants were recovered only from calluses with normal DNA content, making cytometric screening a predictor of regeneration competence rather than a *post hoc* check (Ochatt et al., 2000). Researchers have since integrated this approach into DNA-free workflows. In four elite winegrape cultivars, including the highly recalcitrant Cabernet Sauvignon, flow cytometry identified and excluded non-diploid regenerants, enabling recovery of edited, non-transgenic diploid clones with normal growth and development within four to five months (Böttcher et al., 2025).

A complementary strategy targets regeneration competence itself. Transient expression of defined combinatorial transcription factors has been reported to reprogram mesophyll protoplasts toward pluripotency, raising shoot regeneration in *Arabidopsis* from around 17% in untransfected controls to approximately 90% and enabling regeneration in species and genotypes that were otherwise recalcitrant, including *Capsicum annuum* and soybean cv. Williams 82; the effect was associated with a shift towards permissive chromatin and sustained expression of pluripotency markers (Jeena et al., 2026, preprint). The reprogramming factors are delivered as plasmids and are absent from the regenerated plants, so the approach yields transgene-free material by a process that is not DNA-free, the same distinction discussed in Section 8.4.3. Whether protein or mRNA cargo can drive such reprogramming and thus be combined with RNP delivery in a fully DNA-free workflow remains unclear.

This does not negate the advantages of single-cell delivery set out in Section 6.1, but it qualifies them. The edit itself is clean and clonal; the genome in which it is recovered may not be. Recovering an edited line therefore requires selection against culture-induced change alongside selection for the intended modification, and the burden falls hardest where the target is an elite cultivar whose agronomic performance is the reason for editing it.

### 7.3 Particle bombardment limitations

Biolistic delivery utilizes gold microparticles that are covered with editing cargo, propelled into plant tissue using compressed gas, providing a method for introducing RNPs straight into cells that maintain their cell walls. Gold particles can bind RNA and proteins, delivering these large molecules into plant cells via bombardment (Imai et al., 2020). However, biolistic delivery is intrinsically unpredictable. Variations can be in penetration depth, distribution of cargo, and the quantity of cells that receive the material, which are significantly inconsistent and challenging to standardize. Additionally, coating RNPs onto metal particles may cause clumping or reduced enzymatic effectiveness under less-than-ideal conditions, and the technique is mainly limited to tissue suitable for bombardment, such as callus or immature embryos. The development of in planta particle bombardment (iPB), which targets the shoot apical meristem (SAM) of seed embryos and eliminates the need for callus culture for regeneration, is a noteworthy advancement in wheat transgene-free editing (Hamada et al., 2017). To enable heritable editing, the iPB technique uses the SAM’s sub-epidermal L_2_ layer, which is destined to develop into germ cells. While iPB-RNP delivery in wheat produced editing efficiencies in the E_0_ generation ranging from 1% to 8.3% (Kumagai et al., 2022), the same method was used to report mutation rates in soybeans ranging from 0.4% to 4.6% across various kinds (Kuwabara et al., 2024). A major drawback of SAM-targeted techniques is the creation of chimera plants, because not all cells in the meristem undergo transformation, even though iPB is a significant step toward editing in intact plant tissues.

### 7.4 Nanoparticle mediated delivery challenges

One of the most promising avenues for delivering cargo into cells with intact cell walls embedded in intact tissue architecture is nanoparticle-based delivery. Due to their small diameter (~0.8 – 1.2 nm), which is significantly smaller than the plant cell wall size exclusion limit of ~20 nm, high aspect ratio, and proven ability to passively cross membranes and cell walls, single-walled carbon nanotubes (SWCNTs) have demonstrated a high gene silencing efficacy in delivering siRNA to intact plant cells. Additionally, the nanotubes protect the RNA cargo from nuclease degradation (Demirer et al., 2020). It has also been demonstrated that double-stranded RNA (dsRNA) may be topically sprayed onto intact plant leaves using layered double hydroxide (LDH) clay nanosheets (BioClay) to induce RNAi without causing tissue damage (Mitter et al., 2017). Despite these developments, several obstacles prevent the immediate use of nanoparticles for DNA-free RNP delivery in crops. The hydrophobic nature of SWCNTs requires surface functionalization with polymers like polyethylenimine (PEI), which can change uptake rates and biocompatibility, and controlling the loading of RNP complexes onto nanotube surfaces is extremely challenging. Uncertainty surrounds the long-term fate of nanotubes in plant cells, including their potential toxicity, biodegradation mechanisms, and accumulation across species and tissue types. Additionally, although CNT-mediated delivery has worked in model systems, it has not yet translated to large-scale, repeatable agricultural applications.

### 7.5 Challenges in cell penetrating peptides

Short, positively charged oligopeptides averaging 6 to 30 amino acid residues, known as cell-penetrating peptides (CPPs), have become a desirable alternative for direct intracellular delivery of genome-editing tools in plants. CPPs can cross cell membranes either independently or in combination with other biomolecules, such as proteins and nucleic acids (Liu et al., 2023). The arginine-rich R9 peptide linked to a reporter protein has been shown to achieve transfection efficiencies of up to 100% in *Arabidopsis* root tips, demonstrating a CPP-mediated, DNA-free transfection method in dicots. Because arginine-rich peptides like TAT and R9 have intrinsic nuclear localization functions that enable the transport of the cargo through nuclear pore complexes, a crucial requirement for genome editing CPPs may therefore be promising vehicles for CRISPR RNP intracellular delivery (Wu et al., 2021). In *Arabidopsis thaliana* callus, a CPP-displayed polygon complex vesicle (CPP-PIC) system has also been shown to transport Cas9 RNP and enable nuclear genome editing. Scaling these technologies to whole plant applications is a significant future challenge, as CPP-mediated transport to intact, differentiated plant tissues is still mainly unknown.

### 7.6 Systemic and tissue specific limitations

A new level of complexity develops when the goal shifts from modifying cells in a leaf disc or embryo to changing specific plant tissues such as vascular tissue, root meristems, or seed endosperm. Current delivery methods provide limited spatial control. Biolistic-based approaches primarily target surface-exposed tissues, whilst nanoparticle carriers have not yet exhibited tissue tropism comparable to viral vectors in animal systems. Protoplast transfection, when paired with optimized regeneration techniques, provides a direct mechanism for introducing CRISPR components, albeit its use is currently confined to species with effective protoplast regeneration systems (Yang et al., 2023). Because no tissue-specific delivery systems exist, achieving homogeneous editing across an organ or within a specified cell population remains a pipe dream. This constraint is particularly troublesome when mosaic editing, in which just a fraction of cells within a tissue are edited, would produce agronomically irrelevant or phenotypically unsatisfactory outcomes.

## 8 Cellular and molecular outcomes of DNA-free genome editing

The cellular and molecular effects of DNA-free genome editing depend not on the delivery method itself, but on the biological reactions that occur once a double-strand break (DSB) is created at the target site. When CRISPR/Cas RNP complexes, *in vitro* transcribed mRNA, or purified proteins are introduced into plant cells, the editing components function temporarily. They facilitate precise cleavage at specific sites, are later broken down by the cell’s nucleases and proteases, and ultimately leave only the genomic scar generated by the cell’s own repair processes (Ramakrishnan et al., 2026; Zhang et al., 2018). Understanding the spectrum of molecular outcomes from the identification and fidelity of the repair products to the frequency of unintended mutations and the inheritance of edits between generations is therefore crucial to evaluating DNA-free genome editing.

### 8.1 DNA repair pathways governing DNA-free genome editing

When Cas9 or Cas12a cleaves both strands of the target DNA, the resulting DSB is only detected and treated by the plant cell’s endogenous DNA repair machinery. In plant somatic tissues, two main mechanisms compete to resolve DSBs. A third, mechanistically distinct route, microhomology-mediated end joining (MMEJ), has been shown to contribute substantially to the range of repair outcomes in plants (Vu et al., 2022). NHEJ is the main pathway for repairing double-strand breaks (DSBs) in plant somatic cells and operates throughout the entire cell cycle without requiring a homologous template. The process begins when the Ku70/Ku80 heterodimer identifies and binds to the broken ends of the DNA, subsequently leading to the recruitment of the DNA-dependent protein kinase (DNA Pkcs), followed by processing by the Artemis endonuclease, and concluding with ligation by the XRCC4-DNA Ligase IV complex (Liao et al., 2024). Because NHEJ rejoins broken ends with little or no sequence information, it usually adds tiny insertions or deletions (indels) at the break site, typically 1 bp insertions or 1–20 bp deletions, which disrupt the reading frame and prevent gene function. When no donor template is available for DNA-free editing, these indels are the predominant mutational consequence. HDR uses a homologous DNA template, either the sister chromatid or an externally introduced donor, to restore the original sequence or implement a specific, intentionally designed modification. In plants, HDR primarily operates during the S and G_2_ phases of the cell cycle, when a sister chromatid is naturally accessible as a template (Liao et al., 2024). Because most plant somatic cells reside in G_1_ or G_0_, HDR is considerably less efficient than NHEJ in vegetative tissues, limiting its use for precise knock-in editing without an external donor (Puchta, 2005). Nonetheless, when a single-stranded oligodeoxynucleotide (ssODN) or a double-stranded donor is provided with the RNP, HDR-mediated precise editing has been demonstrated in various plant species, albeit at a much lower frequency than NHEJ-mediated indel formation (**Figure 3**).

**Figure 3:**
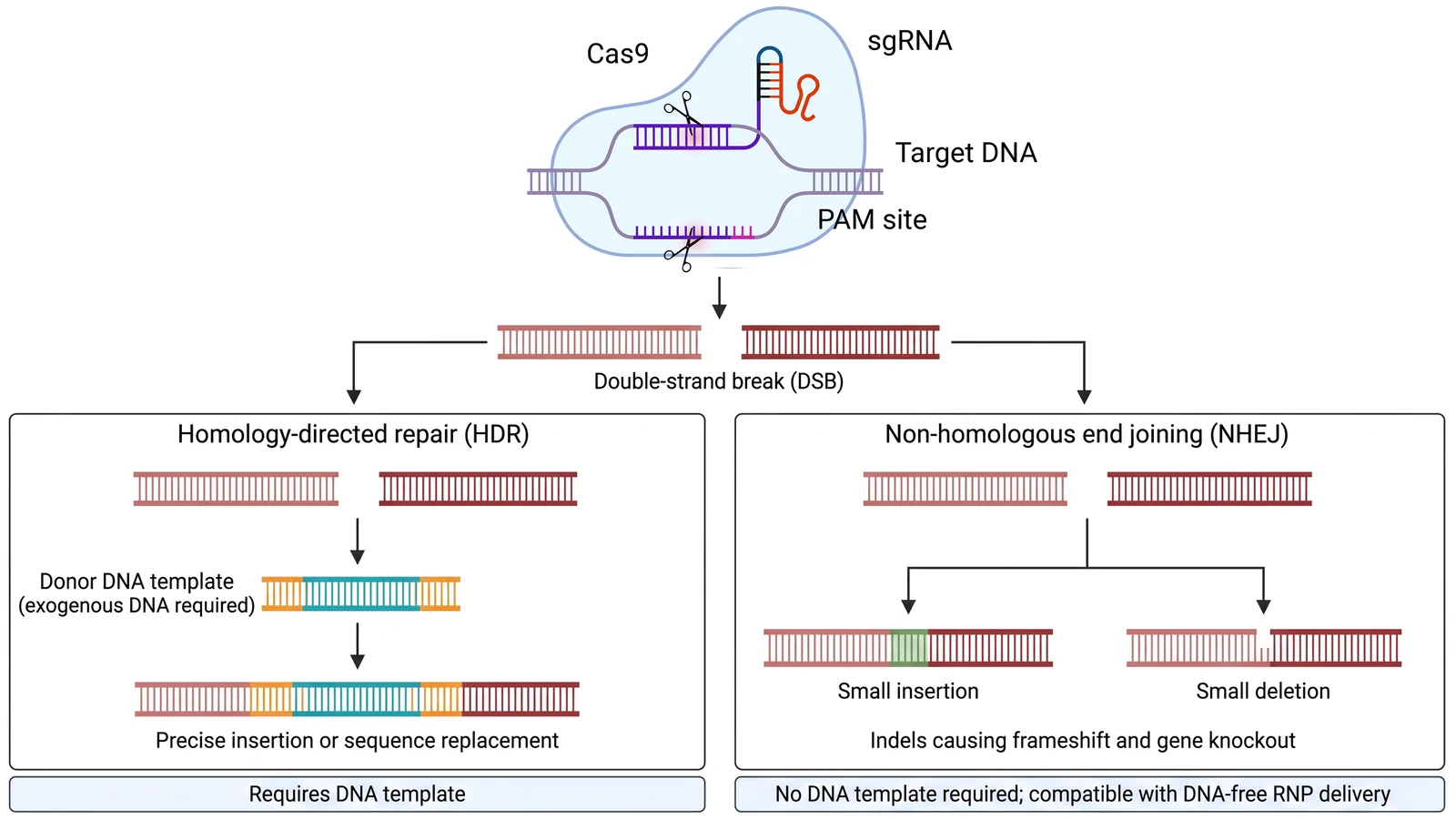
Mechanism of CRISPR/Cas9 genome editing and the repair pathways determining editing outcome. The Cas9 nuclease, guided by a single-guide RNA (sgRNA), binds a genomic target adjacent to a protospacer-adjacent motif (PAM) and generates a double-strand break (DSB) through the coordinated action of its two nuclease domains. One of two principal repair pathways resolves the DSB, and the choice between them determines the edit. Homology-directed repair (HDR) uses a supplied donor template to introduce defined sequence changes, enabling precise insertion or replacement, but requires delivery of donor DNA and is generally inefficient in somatic plant cells. Non-homologous end joining (NHEJ) rejoins the broken ends without a template, producing small insertions or deletions (indels) that frequently disrupt the reading frame and generate loss-of-function alleles. NHEJ predominates in plant cells and is the pathway on which most DNA-free editing depends, since knockouts can be generated from a protein–RNA cargo alone. HDR, by contrast, requires a DNA template and therefore conflicts with a strictly DNA-free workflow, a constraint discussed in Section 8.1. Created in BioRender. Kanagarajan, S. (2026) https://BioRender.com/t8s02ir.

MMEJ serves as an alternative end-joining mechanism that uses short segments of microhomology, typically ranging from 5 to 25 base pairs around the double-strand break (DSB), to join the broken ends before ligation. In contrast to traditional NHEJ, MMEJ functions independently of the essential Ku-Ligase IV components and instead depends on end resection carried out by the MRE11 nuclease, the pairing of exposed microhomologous regions, flap removal, gap-filling synthesis, and final ligation performed by Ligase III (Vu et al., 2022). MMEJ always deletes the intervening sequence between microhomology repeats, resulting in greater and more predictable deletions than traditional NHEJ. Because of its relative predictability, MMEJ has recently been used for precision gene substitution in plants via techniques such as PREMJ, in which short MMEJ donor templates carrying desired mutations, flanked by microhomology arms, are delivered with CRISPR reagents. The local sequence context, the amount of end resection, and the cell cycle stage all influence the relative contribution of MMEJ and classical NHEJ to the spectrum of indels at a particular target locus (Vu et al., 2022).

### 8.2 Editing efficiency and target specificity

The efficiency of editing in DNA-free systems is assessed by the percentage of target alleles that have been successfully modified or contain an indel at the designated locus. In plant protoplasts, RNP-mediated editing efficiencies range from less than 1% to more than 40%, influenced by species, Cas protein type, guide RNA design, RNP dosage, and editing temperature. While Cas9 typically performs better than Cas12a across various temperature conditions, Cas12a remains important for targeting loci without an NGG PAM. In whole tissues, biolistic delivery generally yields lower editing efficiencies, as only a small proportion of bombarded cells receive enough cargo (Kuwabara et al., 2024).

Target specificity is mostly determined by the complementarity of the guide RNA to its genomic target. Mismatches within the seed region (8–12 nucleotides close to the PAM) significantly limit Cas9 activity, whereas mismatches beyond this region may still allow for low-level off-target cleavage (Guo et al., 2023). The temporary nature of RNP administration improves specificity by restricting Cas9 activity as compared to plasmid-based methods that maintain nuclease expression (Ramakrishnan et al., 2026). Cas-OFFinder and CHOPCHOP are computational techniques that aid in off-target prediction, but sequencing-based confirmation is still required (Bae et al., 2014).

Guide RNA design plays an important role in determining editing efficiency and specificity. Chemical synthesis and *in vitro* transcription can both produce guide RNAs (Liang et al., 2017). Chemically produced guide RNAs offer better quality control, more customizable nucleotide alterations, and lower RNase contamination (Hendel et al., 2015). Structural adjustment of the targeting sequence and scaffold region can improve Cas9 activity and target recognition (Chen et al., 2019; Jinek et al., 2012). Chemical modifications like 2′-O-methyl and phosphorothioate groups boost RNA stability and editing performance, with studies showing two- to three-fold increases in mutation frequency compared to unmodified guide RNAs (Hendel et al., 2015; Woo et al., 2015).

### 8.3 Off-target effects and genome integrity

Off-target mutations, including unintended insertions, deletions, or substitutions at non-target genomic sites, remain a significant issue in CRISPR-based crop improvement. These heritable changes can lead to phenotypic and ecological effects. A review of 107 plant genome-editing studies found no detectable off-target modifications in 63% of cases. In contrast, 26% of studies that used direct detection methods found some level of off-target activity (Metje-Sprink et al., 2019). Most of the detected changes were small indels ranging from 1 to 22 base pairs or single-nucleotide substitutions, while large deletions over 100 base pairs were uncommon. Whole-genome sequencing (WGS) is the most reliable method for finding off-target mutations. WGS analysis of 34 Cas9-edited rice lines across T_0_ and T_1_ generations showed no genuine off-target mutations in T_1_ plants (Tang et al, 2018). Studies on grapevines reported only one confirmed off-target indel by WGS and Sanger sequencing. These results emphasize the high specificity of CRISPR/Cas9 in plants when guide RNAs are designed carefully. Delivering DNA-free RNP reduces the risks of off-target effects because endogenous nucleases quickly break down RNPs. This limits Cas9 activity and reduces cleavage at low-affinity off-target sites (Zhang et al., 2018). However, co-delivery of selectable marker plasmids can lead to unintended DNA integration. In rice, biolistic RNP/DNA co-delivery caused random plasmid insertions at target sites in over 14% of cases (Banakar et al., 2019).

### 8.4 Chimerism, mosaicism and heritability of edits

Mosaicism arises when the editing reagent remains active across successive cell divisions, so that cells within a single regenerant carry different repair outcomes. RNP delivery is structurally less prone to this than construct-based delivery. No *Cas9* gene is introduced, so the protein cannot be replenished once the delivered pool is consumed (Woo et al., 2015) and cleavage is correspondingly front-loaded. In mammalian cells, nuclear Cas9 RNP delivered by electroporation remains at a stable concentration through the first 36 h, followed by more than 75% decline by 48 h. However, DNA cleavage and subsequent on-target editing largely completed before substantial depletion of nuclear Cas9 RNPs (Karp et al., 2025). Repaired sites are additionally refractory to re-cleavage, since the indel typically disrupts the protospacer or PAM. Where protoplasts do not undergo a first division for several days after transfection, repair outcomes are therefore fixed before any division occurs, and the regenerant is clonal for a single genotype.

Three qualifications apply. First, these measurements come from mammalian cell culture; equivalent time-course data for Cas9 RNP persistence in plant protoplasts have not been reported. The assumption that editing concludes before the first division currently rests on the interval between transfection and division rather than on direct measurement of nuclease decay in plant cells and is a testable but untested premise. Second, the argument holds only for delivery into single cells. Where RNPs are delivered to multicellular explants or meristems by bombardment or nanoparticle carriers, only a fraction of cells receives the cargo, and mosaicism remains an expected outcome. Third, clonality applies to the edited locus and lineage, not the whole genome; Section 7.2 discusses culture-induced instability affecting other regions.

When CRISPR editing occurs after the first cell division and results in mixed genotypes in plants, trait expression can be complicated, and screening plants to identify transgenics requires laborious downstream analysis (Impens et al., 2023; Scintilla et al., 2022). A frequent problem in grapevine is the generation of chimeras in the transformation process, which is caused by the variability of cutting efficiency and repair mechanisms among the cell populations (Scintilla et al., 2022). In maize, heterologous mutations or genetic mosaics are most frequently found in primary CRISPR transformants, while a genetically stable homozygous line is necessary for functional analysis (Impens et al., 2023). Clinical applications of human germline editing face two major challenges: mosaicism and loss of heterozygosity (Bekaert et al., 2022). These problems are well known and are major limitations in both plant and animal genome-editing systems (Jayarajan et al., 2025; Mclean et al., 2020). Chimerism can be eliminated at the outset of regeneration using single-cell techniques, such as protoplast transfection with CRISPR RNPs, because each cell in the regenerated plant is a progeny of a single edited progenitor. In grapevine, this single-cell strategy, combined with RNP delivery without DNA, produced non-chimeric edited plants for disease susceptibility genes (Scintilla et al., 2022). Regeneration capacity can be enhanced with the development of transformable wheat morphogenic genes, and for genotype-independent transformation, the controlled expression of these genes will significantly reduce chimerism in wheat edited with CRISPR (Kowalik et al., 2026). Delivering CRISPR earlier in development also reduces mosaicism, as edits are made before the first zygotic division.

#### 8.4.1 The mechanisms of action of germline-specific promoters

To overcome this limitation of constitutive promoters, researchers use tissue-specific and inducible promoters for spatiotemporal genome editing without affecting other tissues (Gondalia et al., 2025). Binding of sgRNAs to mobile RNA components like Flowering Locus T (FT) mRNA moves editing complexes to apical meristems, yielding higher editing efficiencies in these tissues than in the initially infected ones (Jacquier et al., 2020). In one generation, the transgene-free, heritable editing of wild-type plants from transgenic rootstocks is realized by introducing gRNA and Cas9 transcripts with graft-mobile tRNA-like sequence (TLS) motifs (Yang et al., 2023). Super-promoter elements (*CmYLCV*), along with promoters active during the sperm-to-zygote transition in haploid induction systems, could enhance editing efficiency and temporal specificity (Li et al., 2025a).

#### 8.4.2 Haploid induction approaches for transgene-free editing

A two-step haploid induction system called “HI-Edit” and “IMGE” was developed to edit elite cultivars that are recalcitrant to transformation, using haploid induction lines and CRISPR/Cas to generate “haploid” progeny lacking the Cas9-containing DNA of the haploid inducer parent, “haploid” lines (Gu et al., 2021; Jacquier et al., 2020). For the maize system, Gu and coworkers reported editing rates > 3% in haploid progenies from five elite inbred lines (Gu et al., 2021), and maternal haploid induction rates of 8.55-20.89% and targeted mutation rates of 0.38-1.46% are reported in 2025 (Li et al., 2025a). Multiplex gene editing, *in vivo* haploid induction and doubled haploid production allow for the production of homozygous lines with various edit combinations within 3 generations without passing through the chimeric, hetero-allelic stage (Impens et al., 2023).

#### 8.4.3 Implications for DNA-free editing

The strategies developed to address mosaicism in tissue-based systems expose a tension within the DNA-free framing. Germline- and meristem-specific promoters restrict nuclease expression to cells contributing to the next generation, while haploid induction systems deliver editing reagents through an inducer parent so that edited haploids are recovered without the inducer genome. Both reduce mosaicism effectively, and both reintroduce DNA: promoter-driven expression requires an integrated cassette, and most haploid inducer lines are themselves transgenic. The resulting plants may be transgene-free, but the process is not DNA-free in the sense used throughout this review. Protoplast-based RNP delivery avoids mosaicism by a different route, through clonal origin rather than through spatial or temporal restriction of nuclease activity and is the only one of these approaches in which no DNA enters the system at any point.

## 9 Applications of DNA-free genome editing in crop improvement

The emergence of DNA-free genome editing has transformed crop improvement by enabling precise genetic changes without incorporating foreign DNA into plant genomes. This technique alleviates regulatory issues linked to transgenic crops while providing unparalleled accuracy in trait development. DNA-free editing technologies have advanced from early protein-based techniques to advanced RNP delivery systems, fundamentally changing contemporary plant breeding.

### 9.1 Vegetable and horticultural crops

To overcome the difficulty of generating transgene-free edited plants in vegetatively propagated and perennial species, the study by (Rocha et al., 2025) combined an efficient Cytosine base editor (CBE) with genetic positive and negative selection in citrus and poplar, using transient *Agrobacterium* delivery of a CBE that co-edited ALS (conferring chlorsulfuron resistance) alongside a gene of interest (*CsNPR3* in citrus, *Pt4CL1* in poplar), together with an FCY-UPP cytotoxin cassette for negative selection on 5-fluorocytosine (5-FC), and a mobile TLS2 RNA motif intended to spread editing into neighboring non-transgenic cells. The strategy was effective in both species, though more so in poplar: in citrus, 24 of 97 regenerants (23.75%) were transgene-free and co-edited at *CsALS* and *CsNPR3, a* ~4.5-fold improvement over prior reports, albeit all chimeric; while in poplar, 15 of 63 shoots (32.5%) were transgene-free and co-edited at *PtALS* and *Pt4CL*1 with the standard vector, including biallelic lines. Unexpectedly, the TLS2 motif reduced editing efficiency and transgene-free recovery in both species (down to 4.86% in poplar), and 5-FC-based negative selection proved unreliable relative to PCR-based screening in both crops. These results establish CBE-based co-editing with positive selection alone as a practical, single-generation route to transgene-free edited citrus and poplar, while highlighting the need for further optimization to reduce chimerism and improve negative selection. DNA-free genome editing has rapidly spread to commercially significant vegetable crops. In tomato, CRISPR RNPs supplied to protoplasts caused heritable SDN-1 mutations in RNA-silencing and pathogen-response genes, imparting disease resistance without chromosomal abnormalities (Ahmad et al., 2026). A DNA-free, multiplexed CRISPR/Cas9 editing system for tomato protoplasts was developed using PEG-mediated delivery of preassembled RNP complexes targeting two strigolactone biosynthesis genes, *CCD7* and *CCD8*, with two sgRNAs each. Protoplasts isolated from Micro-Tom cotyledons and leaves were transfected with four RNP complexes and cultured into green calli, with editing confirmed by fragment analysis and Sanger sequencing. Among 50 screened calli, mutation frequencies reached 30% for *CCD7* and 90% for *CCD8* (26% and 72% biallelic, respectively), with simultaneous editing of both genes achieved in 26% of calli, reflecting stronger activity of the CCD8 guides (Nicolia et al., 2021). These results demonstrate that tomato protoplasts support efficient multiplexed, transgene-free CRISPR/Cas9 editing via RNP delivery, avoiding stable DNA integration and associated regulatory concerns; however, since editing was confirmed only at the callus stage, reliable regeneration of whole plants from edited protoplasts remains the key challenge for translating this system into usable edited tomato lines (Nicolia et al., 2021). Woo and his associates used CRISPR/Cas9 RNPs to disrupt the phytoene desaturase (PDS) gene in lettuce protoplasts, achieving mutation rates up to 46%. This study confirmed the feasibility of RNP delivery to leafy vegetables and established techniques for regenerating edited plants from protoplasts (Woo et al., 2015). Stelmach-Wityk et al. (2024) optimized the protocol and generated protoplasts with an average of 2.5×10^6^ cells/g fresh weight (genotype-dependent) and high viability (88.2%) with the complete resynthesis of the cell wall achieved by 72 hours and calcium alginate embedding. Plating efficiency improved from 46% (day 5) to 62% (day 15), and all cultivars formed microcallus by week 4. Shoots were regenerated from six of seven cultivars, with the highest efficiency obtained in 1:1 cytokinin/auxin medium (NAA, 2iP, low GA_3_) plus reduced sucrose and mannitol, while 2iP alone produced the most callus formation without regeneration, and ‘Haco’ did not regenerate although it formed callus. Andersson et al. (2018) investigated the use of preassembled CRISPR/Cas9 genome editing complexes containing Cas9 and CRISPR RNAs to target the *GBSS* gene that encodes granule-bound starch synthase in the biosynthesis of amylose in potato. It was expected that complete disruption of all four *GBSS* alleles would lead to the production of a high amylopectin, or “waxy“ starch phenotype. The study compared RNPs assembled with either synthetic or *in vitro*-transcribed RNA, using plasmid DNA-mediated delivery as a control. Mutations were generated in up to 9% of regenerated shoots from synthetic-RNA RNPs, and all confirmed mutants were transgene-free. *In vitro*-transcribed RNA RNPs had higher mutation rates (around 22-25%) but also showed unwanted DNA insertion at the target site, possibly due to residual RNA-transcription templates or endogenous potato DNA. In about 2-3% of the regenerated shoots, all four *GBSS* alleles were disrupted, leading to the loss of GBSS activity and the desired high amylopectin phenotype. Overall, the study shows that editing DNA-free tetraploid potato plants with multiallelic CRISPR/Cas9 is feasible and that the plant’s RNA-production strategy can affect editing efficiency and the number of unintended insertion events.

PEG-mediated protoplast transfection of preassembled CRISPR RNPs resulted in DNA-free edits in *Brassica* crops, lettuce, and wild tetraploid tomatoes, with no foreign DNA integration (Ahmad et al., 2026). RNP-mediated editing of the *CaMLO2* susceptibility gene in pepper improved resistance to both biotrophic and hemibiotrophic diseases (Ramakrishnan et al., 2026). In carnation, targeting the anthocyanidin synthase gene altered blossom color (Ramakrishnan et al., 2026). **Table 2** provides a comprehensive overview.

**Table 2:** Applications of DNA-free CRISPR genome editing in vegetable crops for improving quality, disease resistance, and other agronomically important traits.

Species	Target gene / trait	Delivery method	Editing efficiency	Major outcome	Limitations / challenges	References
Cultivated tomato	*SP*, *SP5G*; flowering and plant architecture	Cas9 RNP protoplast editing	31.8% of regenerated plants; mutations were detected in up to 60% of regenerated plants in at least one allele	Regenerated transgene-free tomato mutants with high mutation rate	Shoot regeneration from transfected protoplasts remained a major bottleneck	(Liu et al., 2022)
Wild tetraploid tomato	*SpProSys*, *SpMlo1;* defense response and powdery mildew resistance	DNA-free CRISPR/Cas9 editing of diploid and tetraploid protoplasts using PEG–Ca²^+^-mediated transfection	45.8% (*SpProSys*) and 63.6% (*SpMlo1*) mutation efficiency with RNPs	Heritable edited diploid and tetraploid regenerants without chromosomal changes detected	Some null mutants were sterile, requiring grafting and WT pollination for seed recovery	(Lin et al., 2022)
Tomato	*ALS*; herbicide resistance	*Agrobacterium*-mediated delivery of a CRISPR/Cas9 cytidine base editor	Up to 71% precise editing; 12.9% transgene-free plants	Herbicide-resistant, transgene-free edited tomato plants	Foreign DNA elimination remains difficult in some systems	(Veillet et al., 2019)
Potato	*ALS;* herbicide resistance	*Agrobacterium*-mediated delivery of a CRISPR/Cas9 cytidine base editor	10% transgene-free plants	First-generation transgene-free edited potato plants	Vegetative propagation complicates removal of foreign DNA	(Veillet et al., 2019)
Sweetpotato	*IbeIF4E*, *IbeIF(iso)4E*, *IbCB;* Potyvirus resistance	PEG-mediated Cas9 RNP delivery into leaf protoplasts	10-20%	genome editing of eIF4E-family genes for developing resistance to potyvirus	Polyploidy and vegetative propagation complicate efficient multi-allelic editing	(Brown et al., 2026)

### 9.2 Cereal crops

RNP-mediated protoplast transfection has enabled DNA-free SDN-1 mutagenesis of *P450* and *DWD1* in rice (Ahmad et al., 2026). In wheat, transgene-free editing of *TaARE1* improved nitrogen-use efficiency, delayed senescence, and increased grain yield across three field locations, while editing SD1 in rice landraces reduced plant height, improved lodging resistance, and increased yield under field conditions (Marone et al., 2023). In annual cereals, recovery of transgene-free edited plants is generally facilitated by segregation through selfing or backcrossing, whereas this is more challenging in perennial crops. Zhang and his associates developed a DNA-free CRISPR/Cas12a RNP delivery system, demonstrated in rice and citrus protoplasts (Zhang et al., 2022). This RNP delivery method achieved nearly complete editing at three of four target sites in rice, with comparably high efficiency in citrus protoplasts; in both cases, it exceeded plasmid delivery of the same reagents. A 1:1 ratio of Cas12a to crRNA proved adequate, while NLSs facilitated effective nuclear entry. The research highlights Cas12a RNPs as a viable option for transgene-free genome editing; however, regenerating edited plants from protoplasts remains a significant challenge. **Table 3** provides a comprehensive overview.

**Table 3:** DNA-free CRISPR-based genome editing in cereal crops: target genes, delivery methods, editing outcomes, and technological significance.

Species	Target gene / tissue	Delivery method	Editing efficiency	Editing outcome	Technological significance / limitations	References
Wheat	*Pi21, Tsn1*, and *Snn5* callus cells	Transient delivery of CRISPR DNA or *in vitro*-transcribed (IVT) RNA	Not reported	T_0_ targeted mutants lacking detectable transgene	IVT RNA delivery was less efficient due to instability	(Poddar et al., 2023)
Maize	*MS26, MS45, ALS2, LIG I;* Immature embryos	Cas9 RNP delivery by particle bombardment	2.4 - 9.7% ; ~10% biallelic	Transgene-free edited maize were regenerated without selection	Cereal protoplast regeneration remains difficult, favoring bombardment workflows	(Svitashev et al., 2016)
Rice	*OsPDS, OsROC5, OsmiR528, OsEPFL9*; Protoplast targets	Cas12a RNP delivery	Nearly 100% at 3 of 4 target sites	RNP editing exceeded plasmid delivery under tested temperatures	Protoplast regeneration remains a major downstream bottleneck	(Zhang et al., 2022)

### 9.3 Oilseeds and legume crops

The first efficient protocol for protoplast regeneration of rapeseed (*Brassica napus* cv. Kumily) was developed by Li et al. (2021). Protoplasts were cultured in alginate discs in MI medium (0.5 mg/l 2,4-D and NAA, optimal for viability), followed by MII medium (1.1 mg/l TDZ, low auxin for division); high concentrations of mannitol (100 g/l) were required initially and gradually reduced. The highest shoot regeneration success was achieved in medium containing 2.2 mg/l TDZ + 0.5 mg/l NAA and 20 g/l sucrose (up to 45% regeneration), and prolonged culture at early stages significantly decreased success. PEG-mediated transfection had an efficiency of 40-80%, and targeting of *BnGTR1/BnGTR2* by the CRISPR/Cas9 system resulted in 30-80% of shoot regeneration and 18% mutation efficiency with no transgene integration, the first transgene-free, protoplast-regenerated edited rapeseed plants useful for the reduction of glucosinolates as an improvement in the quality of seedcakes (Li et al., 2021). This route has since been demonstrated at scale in oilseed *Brassica*: Sahab et al. (2024). regenerated and screened 525 shoots from RNP-transfected mesophyll protoplasts; 62% carried one or more mutated target alleles and 50% carried biallelic mutations at both targeted loci. The same protoplast system was used to test guide RNA efficacy before committing to regeneration, illustrating its dual role as a screening and production platform. In a separate study, DNA-free CRISPR/Cas9 RNPs were delivered into leaf protoplasts of rapeseed (*Brassica napus* cv. Kumily) to target seed-expressed *bnax.sgt.a* paralogues encoding a key enzyme involved in sinapine biosynthesis. Editing efficiencies of 28-36% were observed across 50 T_0_ shoots tested, with stable, transgene-free biallelic knockout lines obtained in subsequent generations. Seed sinapine content was significantly and heritably reduced, falling on average from 8.64 to 5.08 mg/g (41%) in T_2_ double mutants, with the best individual plant reaching 3.92 mg/g (49% reduction), and without detectable penalties to growth, flowering, fertility, or seed weight. While this confirms the feasibility of protoplast-based CRISPR RNP editing to generate transgene-free, low-sinapine rapeseed, the best result still fell short of the ~2 mg/g target for unrestricted feed use, suggesting further gains may be possible by screening additional lines (Moss et al., 2025).

The oilseed *Brassica* glucosinolate system illustrates where DNA-free delivery currently stops. The precedent set by (Li et al., 2021) in this crop was transgene-free but partial, disrupting neither gene family completely. Family-complete knockouts of the transporter and regulatory genes governing this trait have been achieved across all homeologs of *GTR1* and *GTR2*, and of *MYB28* and *MYB29*, in hexaploid *Camelina sativa* (Hölzl et al., 2023), and across multiple *GTR* homologs in *B. juncea* (Mann et al., 2023), but in each case by stable transformation, leaving a transgene to be segregated away. The constraint is therefore delivery rather than target biology. The closest published approach in this crop, described above, is the DNA-free knockout of *BnSGT.a* paralogues in cv. Kumily (Moss et al., 2025). That work shows that RNP delivery to rapeseed protoplasts can produce stable, agronomically sound edited lines in an elite background; it does not show whether the same route scales from a small set of paralogues to a gene family spanning two subfamilies and twelve copies. Whether RNP delivery can disrupt all twelve members of the *GTR1*/*GTR2* family in a single generation, in an elite background, and yield regenerants free of integrated DNA has not been reported. This is where the multiplex design considerations of Section 10.1 and the regeneration bottleneck of Section 7 converge, defining the principal outstanding test of protoplast-based DNA-free editing in oilseed crops. The regeneration step compounds the difficulty, since culture-induced variation must be selected against while the intended edits are selected for (Section 7.2).

Targeting genes responsible for erucic acid and polyunsaturated fatty acid (PUFA) biosynthesis, namely *FAE1*, *FAD2* and *ROD1*, in the field cress (*Lepidium campestre*), a promising Nordic oilseed crop, was achieved by protoplast-based CRISPR/Cas9 editing. The *FAE1* knockout significantly increased oleic acid content from 13% to about 30% and nearly eliminated erucic acid in the oil, while the *ROD1* knockout produced less dramatic changes, with oleic acid increasing to about 23% and PUFA content reduced. The most significant gains came from crossing *fad2* knockout with heterozygous *fae1* disruption, yielding lines with up to 66% oleic acid, significantly lower PUFA content, and low erucic acid. Overall, these findings show that the CRISPR/Cas9 system can quickly alter the fatty acid profile of field-cress for a healthier crop, potentially enabling its domestication as an oilseed crop for food, feed, and biofuel applications (Sandgrind et al., 2023). For soybean, the delivered RNP complexes to protoplasts by PEG were found to have a mutation rate of 4.2-18.1% at the *GmCPR5* genome site, with a major limitation being the regeneration of whole plants from edited protoplasts (Subburaj et al., 2022). The efficiency of Cas12a RNP-based protoplast editing ranged from 0 to 17.5% for soybean target genes and nuclease subtypes (Zhang et al., 2021). Subburaj and Agapito-Tenfen in 2023 established targeted mutagenesis in soybean protoplasts by electro-transfection of RNP complexes, extending DNA-free delivery in a species in which protoplast regeneration remains challenging (Subburaj and Agapito-Tenfen, 2023).

To overcome the transgene integration and tissue-culture bottlenecks, the iPB-RNP platform combines DNA-free editing with genotype-independent, regeneration-free recovery of the edited soybean plant (Teng, 2024). This proof of concept has been expanded to a dicot crop plant, demonstrating more potential applications for dicotyledonous species (Teng, 2024). In addition to DNA-free editing strategies, gene-edited soybean has also advanced towards its commercialization. A soybean variety developed with high-oleic and more stable oil was the first gene-edited food product to be commercialized in the United States (Nerkar et al., 2022).

### 9.4 Perennial tree crops

DNA-free genome editing has emerged as a promising approach for improving perennial tree crops. These plants usually have long generation times, high levels of genetic variation, and face challenges with traditional genetic modification. This method introduces genome-editing tools like CRISPR/Cas9 RNP complexes directly into plant cells without using any external DNA. This enables precise genetic modifications while preserving the valuable genetic traits of perennial varieties. This technique is particularly useful for fruit and forestry trees propagated clonally, since breeding to separate transgenes is not feasible. In citrus, LbCas12a RNP delivery into protoplasts derived from embryogenic callus of Hamlin sweet orange achieved 90.8% editing at the *CsPH5* locus at 0.1 µM RNP, falling to 40.1% at 0.01 µM, whereas plasmid delivery of the same reagents gave nearly undetectable activity; regeneration of whole plants from these protoplasts was not achieved and remains to be established (Zhang et al., 2022). A short-term chemical selection strategy involving 3-day transient kanamycin treatment increased transgene-free editing efficiency by 17-fold in citrus, improving mutant shoot recovery from 0.017% to 0.291% (Li et al., 2025b). In poplar, microparticle bombardment enabled regeneration of gene-edited trees free of Cas9 and antibiotic-resistance transgenes, although frequent donor-DNA integration at Cas9-induced break sites was observed (Hoengenaert et al., 2023). In sterile, vegetatively propagated banana, where transgene segregation is not feasible, an *Agrobacterium*-mediated co-editing strategy coupled with embryogenesis and chlorsulfuron selection generated transgene-free plantlets carrying mutations at multiple target sites, which were confirmed by whole-genome sequencing (Van Den Broeck et al., 2025). For instance, researchers have successfully used RNP-mediated DNA-free genome editing in grapevine (*Vitis vinifera*). In this case, researchers induced specific mutations via protoplast transfection without incorporating exogenous DNA into the genome (Najafi et al., 2023). Researchers have also investigated DNA-free CRISPR editing technologies in raspberry (*Rubus idaeus*). These technologies enable modifications to genes associated with key horticultural traits while preserving cultivar identity (Creeth et al., 2025). In forest tree species such as larch, particle bombardment to introduce Cas9-sgRNA RNP complexes into embryogenic callus has facilitated the regeneration of edited plants through a wholly DNA-free editing methodology (Ma et al., 2024). These methods help develop traits in perennial crops that increase disease resistance, improve fruit quality, increase stress tolerance, and enhance growth. They also help transgenic plants avoid legal issues. Overall, DNA-free genome editing is a powerful tool for accelerating breeding programs and functional genomics research in perennial tree crops. This will lead to long-lasting improvements in agriculture. **Table 4** provides a comprehensive overview.

**Table 4:** Representative applications of DNA-free CRISPR genome editing in perennial fruit and forest tree species, highlighting target genes or tissues, editing methods, major outcomes, and the species-specific challenges addressed.

Species	Target gene / tissue	Editing method	Editing efficiency	Major outcome	Limitations / challenges	References
Grapevine	*GFP*; embryogenic callus protoplasts	Cas9-sgRNA RNP transfection	Not reported	Regenerated whole edited grapevine plants after DNA-free editing	Earlier gap was combining protoplast transfection with whole-plant regeneration	(Najafi et al., 2023)
Grapevine	*MLO-7*; protoplasts	Cas9 RNP PEG delivery	0.1%	Susceptibility gene editing for powdery mildew research	Regeneration from edited protoplasts remained limiting in woody crops	(Malnoy et al., 2016)
Apple	*DIPM-1, DIPM-2*, and *DIPM-4*; protoplasts	Cas9 RNP PEG delivery	0.5-6.9%	Fire blight susceptibility targets edited without DNA integration	Woody species show low regeneration efficiency and delivery difficulty	(Malnoy et al., 2016)
Raspberry	*PDS, PG, WRKY52, NPR1*; protoplasts	Cas9 RNP transfection	19% for PDS	First DNA-free RNP editing platform in raspberry protoplasts	Protoplast isolation is a barrier, and gRNA performance was highly variable	(Creeth et al., 2025)
Poplar	*CCR2*; Regenerable tissues	Microparticle-mediated transient delivery	Up to 3.0% transgene-free lines	Edited poplar lines free of Cas9 and selectable-marker transgenes	Frequent donor/plasmid fragment insertion at DSBs complicates strictly DNA-free framing	(Hoengenaert et al., 2023)

## 10 Emerging trends in DNA-free genome editing

DNA-free genome editing is more than just gene modification; it is sophisticated crop improvement. Precise base editing, which allows targeted editing of individual nucleotides without altering overall gene function, is a major advance. This capability lets researchers alter enzyme properties, such as substrate preference or thermal stability. Researchers have edited enzymes involved in starch biosynthesis in crops such as wheat and maize to produce starches with desirable industrial and food-processing characteristics. Multiplexed base editing, which applies multiple base editors with different PAM or base-conversion preferences, enables the concurrent introduction of multiple point mutations in a single transformation event. This strategy is especially useful for polyploid crops like wheat and cotton, where multiple homologous gene copies must be modified for uniform trait expression.

Another important development is tissue-specific and inducible genome editing systems. Most existing inducible systems are based on DNA delivery, but efforts are underway to control RNP activity using light-activated Cas9 variants. These systems can enable precise temporal control of editing, potentially reduce mosaicism and improve editing consistency during plant regeneration. In addition, epigenome editing using catalytically inactive Cas9 fused to epigenetic modifiers has been demonstrated through RNP delivery, enabling transient regulation of gene expression without permanent DNA sequence changes. This strategy has been used to analyze gene function and to optimize the expression levels of agronomically important genes, especially in crops where stable transformation is still a challenge. RNP delivery is advantageous for applications needing short-lived changes in gene expression during specific developmental stages. Sequential RNP delivery has also allowed for gene stacking to accumulate multiple desirable traits within elite cultivars. By introducing different RNP complexes targeting different loci, researchers have created crops with combined traits such as herbicide resistance, disease tolerance, and improved quality characteristics through iterative rounds of protoplast transformation and regeneration. This method is particularly advantageous for species in which conventional breeding would require several generations of backcrossing to combine traits from different genetic backgrounds.

### 10.1 Multiplex gene editing

Multiplex genome editing involves editing multiple genes or genomic locations in a single transformation. This approach is especially valuable for enhancing crops, where complex traits such as yield, stress resistance, and quality involve multiple genes. DNA-free genome editing with RNP complexes enables efficient multiplex editing without inserting foreign DNA into the plant genome. Researchers multiplex-edited rice genomes using polycistronic tRNA-sgRNA arrays. This allowed targeting up to eight gRNAs in a single transformation. Although the work initially used DNA-based plasmids for delivery, later advances replaced multiplex strategies with DNA-free strategies (Xie et al., 2015). According to Zhang and associates, multiplex gene editing using CRISPR/Cas9 RNP complexes was possible, enabling simultaneous editing of genes associated with grain quality, disease resistance, and agricultural performance (Zhang et al., 2018). Two design strategies underlie DNA-free multiplexing, and they scale differently. The first is to increase the number of distinct RNP complexes delivered; because guides are supplied as reagents rather than as expression cassettes, adding targets requires no additional construct assembly, and stoichiometry can be adjusted independently for each guide. Klimek-Chodacka et al. (2021) delivered three RNPs simultaneously to carrot protoplasts, achieving overall editing of up to 71%, with deletions of 33–152 nt generated between target sites at frequencies up to 12%. The second strategy exploits sequence conservation between homoeologs, using a single guide to cleave many copies at once; (Yang et al., 2017) showed that sgRNAs directed at conserved regions of paralogous genes in *B. napus* produced mutation frequencies of 27.6% to 96.6% across copies. In polyploids, the second strategy scales considerably more efficiently, since the number of guides required is set by the degree of sequence divergence within the family rather than by the number of gene copies. Both strategies share a liability: simultaneous cleavage at multiple sites can generate inter-site deletions, inversions, and more complex rearrangements that routine amplicon genotyping does not detect, so multiplex editing in polyploids requires verification methods that can resolve individual homoeologs and structural outcomes.

It has also been used in crop domestication studies by multiplex editing. Zsögön and colleagues demonstrated the domestication of wild tomato (*Solanum* sp.*)* quickly by editing six domestication-related genes that regulate plant structure, fruit size, and fruitfulness (Zsögön et al., 2018).

### 10.2 DNA-free base editing

Base editing is an important improvement in genome editing technologies. It enables accurate nucleotide modification without generating a double-strand break. Base editors are fusion proteins in which a catalytically impaired Cas protein, usually a nickase, is joined to a nucleotide deaminase. This complex allows direct conversion of particular bases in the genome. Shimatani and his coworkers in 2017 first demonstrated cytosine base editing in crop plants using a CRISPR/Cas9-cytidine deaminase fusion, obtaining efficient C-to-T conversions with low indel formation and heritable substitutions without a donor DNA template, although delivery in that study was construct-based rather than DNA-free. The technique enabled the introduction of premature stop codons and amino-acid replacements without donor DNA templates (Shimatani et al., 2017). Subsequently, researchers extended editing with adenine base editors (ABEs) that enabled A-to-G modifications. Li and colleagues delivered an ABE construct (a Cas9-adenosine deaminase fusion) into rice and wheat to introduce specific amino acid substitutions, conferring herbicide resistance in rice via *OsACC* editing as well as altering key yield-related loci (Li et al., 2018). Cytosine and adenine base editors can be used to facilitate precise modification of target bases in crop genomes. Researchers have successfully delivered base-editor constructs in *Brassica napus* (canola), producing targeted base substitutions with minimal off-target mutations (Kang et al., 2018).

### 10.3 Prime editing without DNA integration

Prime editing is the most powerful known genome editing technology. It enables all twelve possible base replacements and small insertions and deletions without producing double-strand breaks or requiring donor DNA templates. Lin et al. (2020) demonstrated prime editing in rice by introducing a prime editor into protoplasts via construct-based delivery. They achieved desired insertions and accurate nucleotide replacements, with editing efficiencies of 5-20% (Lin et al., 2020). Although this was less efficient than conventional CRISPR/Cas9 editing systems, prime editing was far more precise for targeted genome changes. In addition, researchers have extended prime editing to other crops. Overall efficiency has improved through advances in pegRNA design, delivery systems, and optimized prime-editor variants, enabling its use to improve more complex metabolic and agricultural phenotypes in crops. A major use is precisely altering enzymes in major biochemical pathways. Precise nucleotide replacements may modify activity or substrate specificity or alter enzyme heat stability without interfering with overall protein function. This same editing has been extended to starch biosynthesis genes in wheat and maize, resulting in starch variants with enhanced functional characteristics for both food and industrial applications. Multiplexed base-editing systems with a variety of PAM specificities can simultaneously edit multiple related genes in polyploid crops. Epigenome editing is another emerging field, in which catalytically inactive Cas9 (dCas9) is fused with epigenetic regulators to temporarily silence or activate genes without altering the DNA sequence. Expressing these components as RNP complexes allows temporary regulation of gene expression, which can be used to investigate gene functions or increase or decrease gene expression during a given growth phase. Research also explores inducible genome-editing systems in which light or chemical signals control Cas9 activity. Most existing inducible systems rely on DNA delivery, but there is a push to update them to DNA-free RNP-delivery systems, which can provide a tight timing window for genome editing with limited mosaicity. Gene stacking has also been facilitated by sequential delivery of RNP complexes such that several valuable qualities such as herbicide resistance, disease and quality traits can be added in elite cultivars without the extensive backcrossing.

## 11 Regulatory landscape, public acceptance and commercialization

Assessment of genome-edited plants varies widely among countries. According to the UK-CGIAR Centre’s Genome Editing Regulations Tracker, most jurisdictions in Africa, the Americas, Asia and Australia now treat genome-edited crops in the same way as conventionally bred crops, whereas a minority, including New Zealand, Madagascar and Thailand, continue to regulate them as GMOs (Europe’s two tiers of genome editing, 2026).

The United Kingdom illustrates that regulatory divergence can occur within a single state. The Genetic Technology (Precision Breeding) Act 2023 applies only in England, establishing a product-based pathway for organisms whose genetic changes could have arisen naturally or through traditional breeding, broadly comparable to the EU’s NGT category 1 classification. Scotland and Wales have not enacted equivalent legislation and continue to regulate genome-edited organisms as GMOs. The English regime is itself subject to legal challenge: in June 2026 the High Court granted permission for judicial review of the decision by the Secretary of State for Environment, Food and Rural Affairs not to require labeling and traceability for precision-bred organisms, on the ground that the decision was irrational (Europe’s two tiers of genome editing, 2026).

The EU has historically been the most restrictive of the major jurisdictions. Under Directive 2001/18/EC and following the 2018 ruling of the Court of Justice of the European Union that organisms obtained by new mutagenesis techniques fall within the scope of GMO legislation, all gene-edited plants, including SDN-1 and SDN-2 products containing no transgene, were subject to full authorization, risk assessment, traceability and labeling. This position has changed. Regulation (EU) 2026/1388, adopted by the European Parliament and the Council on 17 June 2026, entered into force on 16 July 2026 and will apply from 17 July 2028; until then, plants obtained by targeted mutagenesis and cisgenesis remain subject to the existing GMO rules (European Commission, 2026a).

The Regulation distinguishes two categories of NGT plants. Category 1 plants are those judged equivalent to natural or conventionally bred plants against technical criteria set out in Annex I; following a verification procedure, these plants and their products are exempt from GMO legislation. Category 2 plants harbor more complex modifications and remain within GMO legislation, but with adaptations: a tailored risk assessment, the possibility of adapted arrangements where analytical identification and quantification methods are not feasible, and the possibility of waiving post-market environmental monitoring. Certain traits, notably tolerance to herbicides and the production of a known insecticidal substance, exclude a plant from category 1 irrespective of the extent of its genetic modification. The Regulation covers only targeted mutagenesis and cisgenesis applied to plants, and NGT plants of either category may not be certified organic (European Commission, 2026a, 2026b).

Three features of the Regulation bear directly on the DNA-free approaches considered in this review. First, DNA-free SDN-1 editing produces precisely the class of change the category 1 pathway was designed to accommodate: RNP delivery yields small indels at a defined target and introduces no foreign sequence, so the Regulation removes what has until now been the principal regulatory obstacle to deploying RNP-based editing in European breeding programs. Second, eligibility is constrained by the number and nature of the changes as well as by the technique, so the multiplex strategies discussed in Section 10.1, and particularly the simultaneous disruption of many homoeologous copies in polyploid crops, engage the Annex I limits in a way that single-locus editing does not. Whether a given multiplex design falls within category 1 is therefore a question to be settled at the guide-design stage rather than after regeneration. Third, any plant engineered for herbicide tolerance or to produce a known insecticidal substance is assigned to category 2 automatically, on environmental grounds, however minor the underlying modification. Several DNA-free applications discussed in Sections 9 and 10, including ALS-based herbicide-tolerance edits used both as traits and as co-selection markers for recovering transgene-free regenerants, would therefore remain subject to full authorization. Eligibility for the simplified pathway depends on the trait, scale, and technique. The Commission’s monitoring program must in turn track the application and effects of this trait-based exclusion, suggesting that its scope will remain contested.

The Regulation’s enforcement architecture reflects this limitation directly. The Commission’s implementation strategy provides for verifying compliance through inspections and documentary checks for all NGT plants, with laboratory testing specified only for category 2. Verification of category 1 status therefore rests on documentary evidence of breeding history rather than product analysis, consistent with the European Network of GMO Laboratories’ assessment that products of targeted mutagenesis and cisgenesis generally cannot be distinguished analytically from conventionally bred material (ENGL, 2023). Enforcement against undeclared imports remains correspondingly difficult, and the Regulation itself acknowledges the organic sector’s exposure: organic producers are not penalized for the accidental presence of category 1 material. Much of the framework’s substantive content will depend on delegated and implementing acts due by the date of application.

According to Ricroch et al., (2026) a worldwide survey of 196 UN countries, 24 countries allow gene-edited crops for any purpose, 37 countries have legislation on the table, and 114 countries have no laws whatsoever. Ricroch states that a country that has previously authorized transgenic crops is 22.6% more likely to authorize gene-editing crops (Ricroch et al., 2026). The Japanese experience shows the potential and conflicts that can arise when deregulation is introduced. In 2019, Japan removed the requirement to label genome-edited organisms that do not contain any exogenous nucleic acids under its Cartagena Law, and approved four varieties of food crops (such as GABA-enriched tomato and waxy corn) as non-LMO (Ishii, 2025). Nevertheless, the tomato is the only plant available to consumers, marketed only online, and consumer awareness remains low; some consumers demand mandatory labelling (Ishii, 2025).

Consumer response appears to depend less on the technique than on its purpose. In a Spanish choice experiment, consumers were more willing to select CRISPR-edited tomatoes when the edit was described as reducing pesticide use, lowering water consumption, or improving nutritional quality, and less willing when it was described as reducing food waste or increasing yield (Ballco et al., 2026). This pattern maps closely onto the structure of the new EU framework: the traits excluded from category 1 status, herbicide tolerance and the production of insecticidal substances, are those most closely associated with the first generation of transgenic crops and least aligned with the environmental and health benefits consumers report valuing. Acceptance of DNA-free editing may therefore depend as much on which traits are selected for early commercialization as on the regulatory classification itself. This bears directly on which of the applications reviewed in Sections 9 and 10 are likely to reach the market first.

A major court case in December 2024 in the United States overturned part of the USDA SECURE rule because the USDA could not provide a reasonable basis for some exemptions, a blow to risk-based, product-based regulation (Venkataraman et al., 2025). The ruling leaves uncertainty over almost 100 GE crops that had previously received exemption determinations (Venkataraman et al., 2025). EU and exporter regulations diverge, posing WTO challenges because detecting non-compliant products is technically infeasible (Bhattacharjee et al., 2023; Sprink et al., 2022). However, the biggest deficiency is the lack of solutions to detect and trace DNA-free edits, which directly weakens the enforcement of any regulatory framework, whether permissive or restrictive (Bhattacharjee et al., 2023; Sprink et al., 2022). Although vegetatively propagated species such as bananas, grapes, and raspberries pose the greatest regulatory challenge, research in these areas is emerging but remains relatively sparse (Creeth et al., 2025; Kato et al., 2026; Van Den Broeck et al., 2025). DNA-free genome-edited crops therefore occupy a regulatory position defined less by their molecular composition than by jurisdiction: scientifically indistinguishable from conventionally bred material yet assigned to different categories under GMO definitions written for a different technology (**Table 5**).

**Table 5:** Regulatory frameworks and the status of DNA-free genome-edited crops across selected jurisdictions.

Jurisdiction	Framework type	Status of DNA-free edits	References
USA	Product-based	SDN-1/SDN-2 deregulated under 2020 SECURE Rule	(Venkataraman et al., 2025)
EU	Process-based, with tiered exemption from July 2028	Regulation (EU) 2026/1388, in force 16 July 2026, applies 17 July 2028. Category 1 (equivalent to conventional under Annex I criteria) exempt from GMO legislation after verification; category 2 remains within GMO legislation with adaptations. Herbicide tolerance and insecticidal-substance production excluded from category 1. Directive 2001/18/EC applies until 2028	(Regulation (EU) 2026/1388; European Commission, 2026b)
Japan	Product-based	SDN-1/SDN-2 treated as non-GMO	(Ishii, 2025)
India	Product-based	SDN-1/SDN-2 exempted (2022)	(Bhattacharjee et al., 2023)
Argentina	Product-based	First to exempt (2015)	(Ricroch et al., 2026)

## 12 Commercial viability and economic considerations

Compared with conventional genetic engineering, the proof-of-concept cost of genome editing is estimated at $2–3 million, significantly less than conventional genetic engineering ($15–18 million) (Kalaitzandonakes et al., 2023). It is suggested that the cost of developing genome-edited crops into a commercialized product over 5 years is an average of $10.5 million (Kalaitzandonakes et al., 2023), but exact details of the costs involved in the DNA-free process are not provided. The lack of expensive GMO-based regulatory processes allows commercialization of these products to be significantly less expensive and allows smaller companies to innovate in these areas (Ahmad et al., 2023). In addition to the cost savings of not having to remove the transgenes in subsequent generations, DNA-free approaches are more sustainable and time-saving alternatives to conventional breeding. However, DNA-free delivery is usually performed at high reagent concentrations or at the expense of cell viability through physical delivery methods, increasing regeneration costs in recalcitrant genotypes (Kim et al., 2026). The main infrastructure gap in breeding programs is that DNA-free workflows are traditionally not scalable in resource-limited settings, and delivery and screening methods that do not require specialised equipment will help ensure the technology is not limited to high-resource areas (Gao et al., 2026). The main reason for acute infrastructure barriers in perennial crops is their long generation times and low regeneration efficiency, which make transgene removal through segregation impractical and require delivery systems tailored to the trees in question (Ramakrishnan et al., 2026).

## 13 Conclusion

DNA-free genome editing is a game-changing crop improvement technology that has the accuracy of the newest molecular biology field and the regulatory and acceptance benefits of not integrating permanent transgenes. Further advances in delivery, the expansion of editing options such as base and prime editing, and the establishment of effective regulatory frameworks will determine how much this technology can help ensure global food security and sustain agriculture. With growing pressures on agricultural systems from climate change and population growth, the promise of creating better crop varieties quickly through DNA-free genome editing offers a powerful solution to emerging challenges in meeting the needs of a society concerned about genetic modification. The technology’s precision and efficiency, coupled with its ability to produce plants indistinguishable from those from traditional breeding, make it a key component of sustainable agriculture in the 21st century. Future studies should focus on improving delivery to recalcitrant species, establishing tissue-specific delivery systems, and developing international regulatory frameworks to promote the responsible use of this powerful technology.
